# Immunomodulatory Role of Neuropeptides in the Cornea

**DOI:** 10.3390/biomedicines10081985

**Published:** 2022-08-16

**Authors:** Sudan Puri, Brendan M. Kenyon, Pedram Hamrah

**Affiliations:** 1 Center for Translational Ocular Immunology, Tufts Medical Center, Tufts University School of Medicine, Boston, MA 02111, USA; 2 Department of Ophthalmology, Tufts Medical Center, Tufts University School of Medicine, Boston, MA 02111, USA; 3 Program in Neuroscience, Graduate School of Biomedical Sciences, Tufts University, Boston, MA 02111, USA; 4 Departments of Immunology and Neuroscience, Tufts University School of Medicine, Boston, MA 02111, USA; 5 Cornea Service, Tufts New England Eye Center, Boston, MA 02111, USA

**Keywords:** ocular immune privilege, neuropeptides, receptors, neuroimmune interactions, ocular surface, cornea, immune cells, antigen-presenting cells, trafficking, kinetics

## Abstract

The transparency of the cornea along with its dense sensory innervation and resident leukocyte populations make it an ideal tissue to study interactions between the nervous and immune systems. The cornea is the most densely innervated tissue of the body and possesses both immune and vascular privilege, in part due to its unique repertoire of resident immune cells. Corneal nerves produce various neuropeptides that have a wide range of functions on immune cells. As research in this area expands, further insights are made into the role of neuropeptides and their immunomodulatory functions in the healthy and diseased cornea. Much remains to be known regarding the details of neuropeptide signaling and how it contributes to pathophysiology, which is likely due to complex interactions among neuropeptides, receptor isoform-specific signaling events, and the inflammatory microenvironment in disease. However, progress in this area has led to an increase in studies that have begun modulating neuropeptide activity for the treatment of corneal diseases with promising results, necessitating the need for a comprehensive review of the literature. This review focuses on the role of neuropeptides in maintaining the homeostasis of the ocular surface, alterations in disease settings, and the possible therapeutic potential of targeting these systems.

## 1. Introduction

Corneal transparency is vital for vision, and there are various anatomical and physiological factors that contribute to the maintenance of corneal transparency. The human cornea consists of three major cellular layers with anterior and posterior limiting laminae among them [1]. The outermost layer is the corneal epithelium, which consists of non-keratinized, stratified layers of epithelial cells with immune cells at the basal layer [2], and the innermost layer is the corneal endothelium, made up of a single layer of specialized cells [3,4]. Between the corneal epithelium and endothelium lies the corneal stroma, which is an avascular collagenous layer interspersed with keratocytes and resident corneal leukocytes (RCLs) [2,5,6,7,8]. The immune cells reside not only in the peripheral cornea as previously thought, but are also located in the central cornea, albeit in part in an immature phenotype [2,7,8,9]. The cornea is the most densely innervated tissue of the body and the corneal sensory nerves, together with RCLs, serve as the sentinels of the cornea [2,7,10,11]. Corneal sensory nerves may be activated by warming or cooling the ocular surface, changes in osmolality, mechanical stimulation, trauma, infections, and a variety of chemical irritants, whereas RCLs detect foreign antigens [7,10,12,13,14]. Several studies have shown a significant structural association and a functional interdependence between immune cells and sensory nerves in the cornea [9,15,16,17,18,19,20]. Since both the corneal nerves and RCLs share the expression of neuropeptides and their respective receptors, crosstalk may occur via the neuropeptide–receptor interaction [17,21,22,23]. Thus, understanding the immunomodulatory role of corneal neuropeptides could lead to the discovery of new therapeutic targets for the maintenance and restoration of corneal transparency in different pathological conditions or after surgical procedures such as corneal transplantation and refractive surgeries. Although recent studies have explored the distribution of neuropeptides and their receptors on the ocular surface [24,25,26], their functional role is yet to be fully understood. This review focuses on the neuropeptides and immune cells in the cornea and examines the functional contribution of neuropeptides in corneal immunity studied so far.

## 2. Corneal Immune Privilege

Ocular immune privilege was first described by Medawar as the phenomenon of the prolonged survival of a skin allograft placed in the anterior chamber of a rabbit eye [27]. Because the allografts were rejected only after neovascularization had developed within the grafts, immune privilege was attributed to the passive mechanism of the immunological ignorance of the antigens within the eye, due to the lack of an antigen exit from the grafts through lymphatics and the lack of an immune cell entrance through blood vessels [27]. Further studies suggested that the immune system was aware of the presence of foreign antigens inside the eye and that ocular immune privilege was also maintained by active mechanisms, including immune tolerance induced from within the eye and the immunosuppressive ocular microenvironment [11,28,29,30].

The cornea is an immune-privileged tissue, as demonstrated by its ability to support the prolonged, and even indefinite, survival of allogeneic corneal grafts. Further, allogeneic corneal grafts demonstrate a prolonged survival time when transplanted in a conventional immunological site, such as under the kidney capsule, as compared with skin grafts [11,27,28,29,30,31]. The major contributors to corneal immune privilege are believed to be the lack of lymphatic and blood vessels, the expression of immunomodulatory factors, the presence of a unique repertoire of resident antigen-presenting cells [29,32], and the expression of Fas-ligand [33]. The major populations of resident corneal antigen-presenting cells include conventional dendritic cells (cDCs) [2,7], plasmacytoid dendritic cells (pDCs) [8], and macrophages (MΦ) [34]. After the discovery of mature (MHCII+) cDCs in the peripheral cornea and limbus [35], subpopulations of immature (MHCII^−^, CD80^−^ and CD86^−^) central and peripheral cDCs were also discovered [2,7]. Immature corneal cDCs, which are unable to sensitize T cells with antigen, are distributed throughout the cornea, whereas mature cDCs are present only in the peripheral cornea [36]. Plasmacytoid dendritic cells (pDCs) are another population of RCL that have recently been found within the anterior stroma and are functionally and phenotypically distinct from cDCs [8,9]. pDCs produce type I interferons (IFNs) and have additional roles in the regulation of immune tolerance, as well as T cell immunity [37,38,39,40,41,42,43,44,45,46,47]. The various subsets of corneal resident macrophages are mostly present in the posterior stroma during the steady state [2,19,34,48,49,50,51,52]. Other immune cell subsets are largely absent from the cornea in the steady state; however, peripheral immune cells are recruited to the cornea in response to acute inflammation/injury, and include neutrophils [53], γδ-T cells [54], memory T cells [55], and natural killer cells [56], whereas CD4+ effector T cells are recruited in chronic inflammation such as in dry eye disease [57].

## 3. Corneal Innervation

The cornea is densely innervated by both sensory and autonomic nerve fibers. The cornea receives sensory innervation mostly from the nasociliary branch of the ophthalmic division and some from the maxillary division of the trigeminal ganglion [10]. Animal studies have shown that the cornea also receives sympathetic innervation from the superior cervical ganglion [58,59] and parasympathetic innervation from the ciliary ganglion [10,60]. 

Previous studies have shown close physical and functional connections between sensory nerves and RCLs in the cornea, leading to a growing interest in understanding the interactions between them [9,19]. Corneal nerves also regulate intracorneal chemotaxis and the homing of leukocytes, which are important in maintaining corneal homeostasis [61,62]. In diabetic mice, the local depletion of cDC alters the density of corneal nerve endings, corneal sensitivity, and delays post-wound nerve regeneration [16]. Similarly, the local depletion of cDCs in primary acute herpes simplex virus (HSV)-1 keratitis in mice results in the severe loss of corneal nerves [63]. Sensory denervation by trigeminal axotomy leads to decreased tear secretion, a loss of immune privilege, and an enhanced cDC migration and motility [64]. While corneal cDCs display minimal motility during the steady state [18,65], following sensory denervation, corneal cDCs greatly increase motility in a random walk fashion [21]. Sensory denervation also upregulates vascular adhesion molecules, thereby promoting leukocyte adhesion, rolling, and sticking and ultimately leading to an influx of bone marrow-derived cells into the cornea [20,21,66]. Recent studies have shown that these mechanisms of neuroimmune crosstalk in the cornea could largely be via interactions between neuropeptides expressed by corneal nerves and their receptors in RCLs [19,21,67,68,69,70].

## 4. Neuropeptides and Their Receptors in the Cornea

Neuropeptides are signaling molecules (3–100 amino acids in length) that mediate a wide range of physiological functions through their receptors. Neuropeptides and their respective receptors are expressed by various other cells, including immune cells, in addition to the nerve fibers in the cornea (Table 1).

The sensory nerves in the cornea express, among other things, Substance P (SP), calcitonin gene-related peptide (CGRP), pituitary adenylate cyclase-activating peptide (PACAP), α-melanocyte-stimulating hormone (α-MSH), and galanin [154,155,156,157,158,159,160]. Besides the classical neurotransmitters, the corneal sympathetic nerves also express serotonin and neuropeptide Y, whereas the parasympathetic nerves contain vasoactive intestinal polypeptide (VIP), met-enkephalin, neuropeptide Y (NPY), and galanin [158,161,162,163,164,165,166]. Other neuropeptides, such as neurotensin, adrenomedullin (AM), somatostatin (SST), brain natriuretic peptide, cholecystokinin, vasopressin, and beta-Endorphin, have also been detected in the cornea, but whether they are expressed by sensory or autonomic nerves has not been clearly demonstrated [165,167,168].

Neuropeptides exert their effects mostly through interactions with their receptors, which belong to the superfamily of G protein-coupled receptors (GPCRs). These contain seven transmembrane domains and are coupled with intracellular heterotrimeric G proteins, which transduce the signal intracellularly (Table 2). 

**Figure 1:**
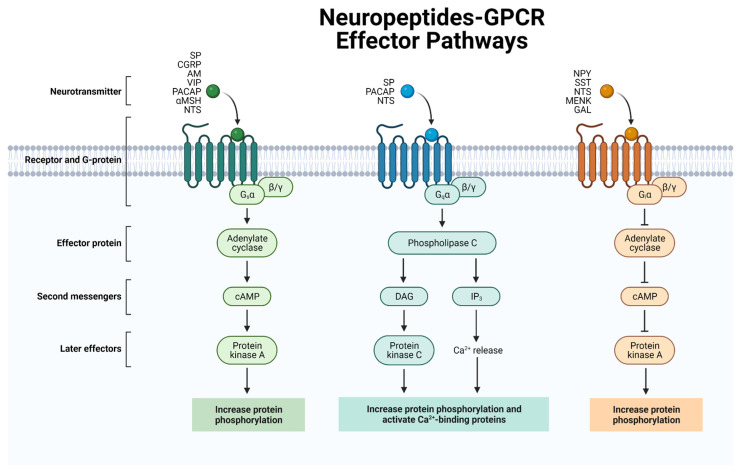
Neuropeptides interact with their G protein-coupled receptors (GPCRs) and the β/γ dimer is separated from the Gα subunit classified as Gs, Gq/11, or Gi/o, which transduce the signal intracellularly via effector proteins. Adapted from “GPCR Effector Pathways”, by BioRender.com accessed on 26 July 2022. Retrieved from https://app.biorender.com/biorender-templates accessed on 12 April 2022. SP—Substance P, CGRP—calcitonin gene-related peptide, AM—adrenomedullin, VIP—vasoactive intestinal peptide, PACAP—pituitary Adenylyl Cyclase activating peptide, NPY—neuropeptide Y, SST—somatostatin.

The heterotrimeric G proteins consist of three subunits—the α, β, and γ subunits. Upon receptor activation, the G protein is activated and the α subunit separates from the βγ dimer. G proteins are classified according to the activity of the Gα subunit as either Gs, Gq/11, or Gi/o (Figure 1).

Gs Signaling: The neuropeptide receptors belonging to the Gs family include the VIP and PACAP receptors (VPAC1R, VPAC2R, and PAC1R), the adrenomedullin and CGRP receptors (AM1R, AM2R, and CGRPR), and two of the tachykinin receptors (NK1R and NK2R).

- Gq/11 Signaling: The neuropeptide receptors belonging to the Gq/11 family include the PACAP receptor (PAC1R) and the tachykinin receptors (NK1R, NK2R, and NK3R).
- Gi/o Signaling: The neuropeptide receptors belonging to the Gi/o family include the neuropeptide Y receptors (NPY1R, NPY2R, NPY4R, and NPY5R) and the somatostatin receptors (SST1R, SST2R, SST3R, SST4R, and SST5R).

### 4.1. Substance P (SP)

SP is a highly conserved prototypical member of the tachykinin family of peptides [213], which also contains other neuropeptides—neurokinin A, neurokinin B, neuropeptide K, and neuropeptide γ [214,215]. Because SP was originally isolated from intestinal extracts, purified and dried in powder form, it was named “Substance P” [216,217]. Although SP was first discovered in equine gut extracts, its homologues have been found in mice, rabbits, and humans, and it is known to be expressed by many other tissues and cell types, including neurons.

#### 4.1.1. Transcriptional Regulation

**Figure 2:**
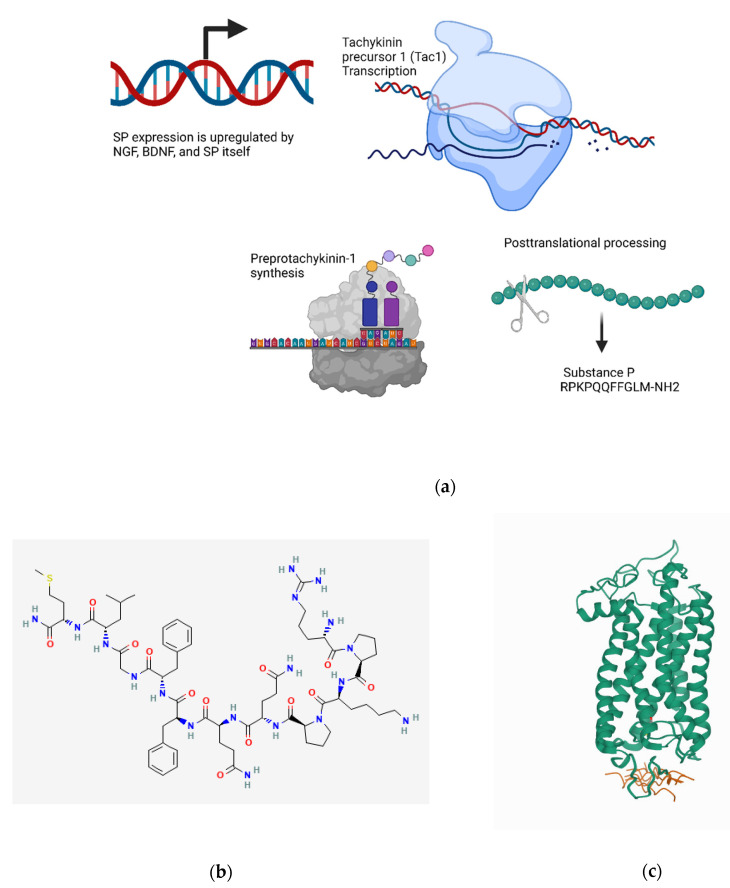
Neuropeptide Substance P and neurokinin receptor: (**a**) transcription and synthesis of SP (created with BioRender.com accessed on 22 October 2021); (**b**) 2D structure image of Substance P (https://pubchem.ncbi.nlm.nih.gov/compound/36511#section=2D-Structure, accessed on 22 October 2021); (**c**) bound-state structure representation of Substance P (brown) to NK1R (green). The solution conformation of Substance P in water was complexed with NK1R. Image from the RCSB PDB (rcsb.org) of PDB ID 2KS9 [73].

SP and neuropeptide K are products of the same gene tachykinin precursor 1 (Tac1), whereas neurokinin B is the product of tachykinin precursor 3 (Tac3). The Tac1 gene contains CRE sites that are bound by ATF2 and CELF, a member of the C/EBP family [218]. Additionally, the Tac1 gene is upregulated by nerve growth factor (NGF) and brain-derived growth factor (BDNF), and Substance P itself may work in an autocrine manner to increase Tac1 expression [71,219]. In vivo studies have revealed that the ECR1 enhancer interacts with the Meis1 transcription factor to control the expression of the Tac1 gene in the amygdala [220] (Figure 2a).

#### 4.1.2. Metabolism and Signaling

The stability of SP depends on enzyme activity, the bound/unbound state, or cellular internalization dynamics. The half-life of SP is longer in plasma (hours) than in tissues (seconds to minutes) [221,222,223]. Unbound SP is hydrolyzed by p-endopeptidase in tissues and by angiotensin-converting enzyme (ACE) in plasma [224]. 

SP coupling with NK1R activates phospholipase C and adenylate cyclase to generate inositol trisphosphate/diacylglycerol (IP3/DAG) and cyclic adenosine monophosphate (cAMP) second messenger systems, respectively [225,226,227]. IP3 increases the level of cytosolic Ca^2+^, DAG activates protein kinase C (PKC), and cAMP activates protein kinase A (PKA). These molecules signal mitogen-activated protein kinases (MAPKK or MEKs), and the expression of cytokines is eventually mediated by the translocation of extracellular signal-related kinase 1/2 (ERK1/2) to the nucleus [228,229,230,231,232,233,234,235,236,237,238]. 

Desensitization starts when SP-bound NK1R is phosphorylated by G-protein-coupled receptor kinases (GRKs), followed by the formation of SP/NK1R-β-arrestin complex and internalization [239]. The exposure to the acidic environment hydrolyzes the phosphate groups from NK1R and releases the bound SP molecule, which is degraded by proteolytic enzymes [240]. Resensitization results from recycling NK1R to the cell surface [241]. 

#### 4.1.3. Immunomodulation and Inflammation

SP plays a substantial role in promoting pain [242,243] and inflammation [244]. SP mediates the recruitment and activation of immune cells by regulating cytokine and chemokine production, such as macrophage inflammatory proteins (MIP-1β or CCL4), MIP-2 or CXCL2, monocyte chemoattractant protein-1 (MCP-1 or CCL2), CCL5, and IL-8 [68,179,228,229,230,231,232,233,234,235,236,237,238,245,246]. SP presumably works mostly through NK1R since the NK1R antagonist can abrogate the recruitment of lymphocytes and monocytes to the inflamed site [232], and NK1R knockout mice have an attenuated chemotactic response of neutrophils [245]. In addition, SP promotes T cell proliferation through the upregulation of IL-2 expression in vitro [247,248,249,250,251], and NK1R knockout mice have reduced T cell proliferation [252]. SP can also modulate the production of cytokines (IL-1, IL-4, and IFN-γ) that induce the expression of NK1R in macrophages [253,254]. The expression of NK1R in T cells is modulated by SP through cytokines IL-12, IL-18, and TNFα, which induce NK1R expression, or IL-10 and TGF-β, which inhibit NK1R expression [255,256,257]. 

Studies have shown that SP:

- Enhances phagocytosis in leukocytes (neutrophils and macrophages) through the stimulation of oxidative burst, synthesis, and the release of reactive oxygen intermediates [258,259,260,261,262].
- Induces macrophages and eosinophils to secrete pro-inflammatory cytokines TNF-α, IL-1β, IL-2, and IL-6 [263,264].
- Promotes mast cell activation via the upregulation of Toll-like receptor (TLR)-2 and the release of histamine and serotonin [265,266], as well as the release of IL-8, TNF-a, and VEGF by increasing the expression of corticotropin-releasing hormone receptor-1 (CRHR-1) [195,267,268,269].
- Enhances NK cell activity and migration [270,271] by upregulating their production of cytotoxic-associated molecules (perforin, granzyme) and natural cytotoxicity receptors (NCR) [272].

#### 4.1.4. Role of Substance P in the Cornea

In the cornea, SP and its receptor, NK1R, are expressed mainly by sensory nerves from the trigeminal ganglion, the corneal epithelium, stromal keratocytes, and immune cells [273,274,275,276]. SP has been detected in normal human tears, which suggests SP’s role in corneal tear film homeostasis [277,278]. SP released from the sensory nerves also induces increased tear secretion [279], and mice with NK1R gene deficiency have reduced basal tear production and develop signs of dry eye disease [280]. SP also regulates the expression of tight junctions (E-cadherin and ZO-1) and inhibits the hyperosmotic-stress-induced apoptosis of corneal epithelial cells in ex vivo cultures [281,282]. Moreover, NK1R knockout mice have an excessive exfoliation of the superficial corneal epithelial cells, indicating the protective role of the SP-NK1R signal transduction pathway [280]. However, a recent study showed that the excessive expression of SP results in accelerated senescence and the exhaustion of residual stem cells, leading to limbal stem cell deficiency (LSCD) [283]. In a preclinical model of LSCD, SP ablation or NK1R blockade significantly increased epithelial wound healing and corneal transparency compared with the wild type [283].

SP-NK1R signaling in the cornea promotes inflammation, nociception, neovascularization, and wound healing [227,284,285,286,287]. During inflammation, SP promotes leukocyte extravasation and chemotaxis [68,179] by inducing the production of IL-1β and chemotactic molecule IL-8 in corneal epithelial cells [179,284,285,288]. The leukocytes recruited in the cornea also contribute to the production of SP and other pro-inflammatory cytokines such as VEGF, TNF-α, IL-1β, IL-8, IL-12p40, IL-23, and IFN-γ [289,290] and have reduced levels of the anti-inflammatory cytokine IL-10 [227]. Through promigratory and angiogenic mechanisms, SP may have a role in the pathogenesis of pterygia [291], allergic conjunctivitis [292], and corneal graft rejection [293]. A correlation of high levels of SP and increased angiogenesis has been reported in neovascularized corneas and in cases of conjunctivitis [284,285]. Similarly, SP/NK1R antagonism suppresses pathologic corneal lymphangiogenesis in DED induced in wild-type C57BL/6 J mice using a controlled-environment chamber without scopolamine [294]. In addition, leukocyte recruitment and cytokine release induced by SP cause an increased severity of herpes simplex virus (HSV) viral [295] and bacterial (*Pseudomonas aeruginosa*) keratitis [288,296]. Besides its pro-inflammatory functions, SP has also been shown to promote corneal wound healing by stimulating the increased synthesis of chemokine IL-8, which enhances the migration of epithelial cells and keratocytes expressing NK1R [287]. In addition, SP has a synergistic action with insulin growth factor-1 (IGF-1) via NK1R to improve the barrier function and attachment of corneal epithelium to the basement membrane during wound healing [297,298]. Taken together, these findings suggest that SP and its metabolites are important factors in maintaining corneal homeostasis and in enhancing wound healing and that SP also promotes inflammation in the cornea. The pro-inflammatory and angiogenic activities of SP can be blocked by using NK receptor antagonists, with potential therapeutic applications.

### 4.2. Calcitonin Gene-Related Peptide (CGRP)

CGRP belongs to the calcitonin family of peptides, comprised of calcitonin, adrenomedullin, adrenomedullin 2 (intermedin), calcitonin receptor-stimulating peptide (CRSP), and amylin [299,300,301]. CGRP was first discovered as a product of the alternative splicing of calcitonin mRNA in the thyroids of aging rats [302]. CGRP is primarily localized to C and Aδ sensory fibers throughout the body and plays a role in pain and vasodilation [303].

#### 4.2.1. Transcriptional Regulation

**Figure 3:**
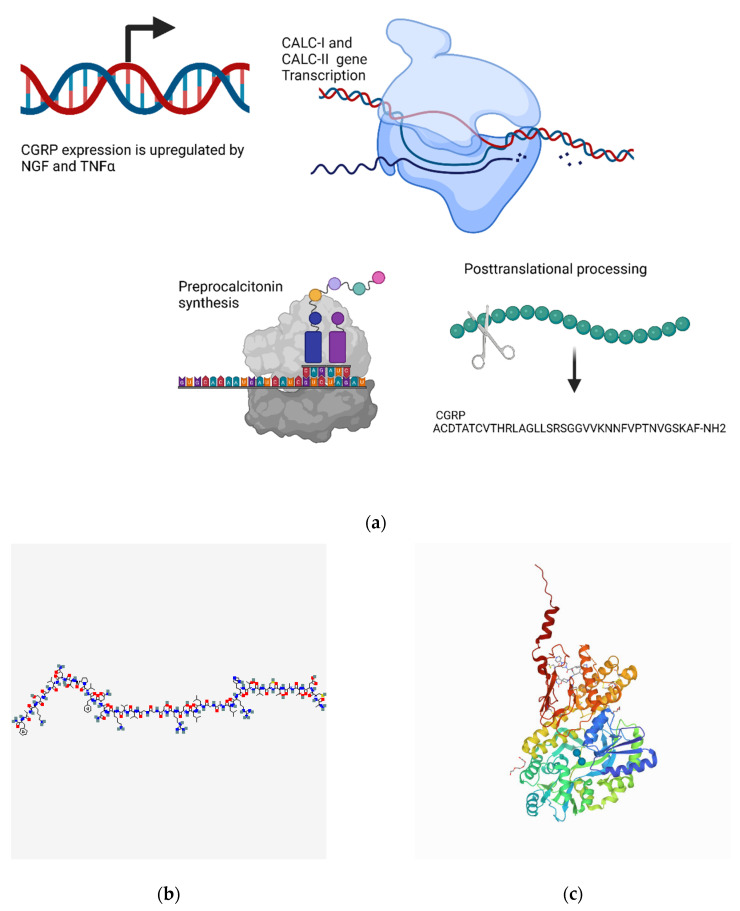
Neuropeptide CGRP and CLR receptor: (**a**) transcription and synthesis of CGRP (created with BioRender.com accessed on 22 October 2021); (**b**) 2D structure image of CGRP (https://pubchem.ncbi.nlm.nih.gov/compound/16132372#section=2D-Structure, accessed on 22 October 2021); (**c**) Crystal structure of a CGRP receptor ectodomain heterodimer with bound high-affinity inhibitor. Image from the RCSB PDB (rcsb.org) of PDB ID 6ZHO [309].

Two distinct genes, CALC-I and CALC-II, produce two forms of CGRP: the CALC-I gene can produce either calcitonin or αCGRP (CGRP-I), and the CALC-II gene produces βCGRP (CGRP-II) [304,305]. The regulation of CGRP production is quite complex and involves many different mediators. The pro-inflammatory molecule tumor necrosis factor (TNF)α activates many signaling pathways, including NF-κB, Jun N-terminal kinase (JNK), and MAP kinase signaling. TNFα has been shown to induce the expression of CGRP, and this effect is thought to be predominantly mediated through the MAP kinase pathway, as indicated by pharmacologic inhibition studies [306]. CGRP transcription is increased following treatment with NGF alone or NGF in combination with activin, which acts synergistically with NGF to promote a further increase in CGRP transcription [307]. Glucocorticoids, such as dexamethasone, have been shown to downregulate CGRP; however, this occurred in only a subset of the cell lines examined, whereas no effect was seen in others. This suggests that additional factors are required for the dexamethasone-induced downregulation of CGRP and that these factors are presumably expressed in a tissue-specific manner [308] (Figure 3a).

#### 4.2.2. Metabolism and Signaling

CGRP is stored in vesicles at the sensory nerve terminal and released by calcium-dependent exocytosis [310,311]. Several mechanisms of the removal or breakdown of CGRP have been proposed: the reuptake of CGRP into the neuron via active transport, the hydrolysis of CGRP by tryptase or endothelin-converting enzyme-1 (ECE-1), or removal by neutral endopeptidase enzyme neprilysin [312,313].

After CGRP receptor binding, the Gαs-dependent stimulation of adenylate cyclase increases the synthesis of cAMP, activating PKA and opening K^+^ channels [314]. CGRP also activates the MAPKs and extracellular signal-regulated kinase 1/2 (ERK1/2). These signaling pathways lead to vasodilation and protect cultured vascular smooth muscle cells from oxidative stress-induced apoptosis [315].

The desensitization of the signals and cAMP responses are attenuated after a second exposure to CGRP due to the activity of PKA and PKC [316,317,318]. After the transient stimulation of the receptor, the CLR is phosphorylated and the β-arrestin complex is internalized to clathrin-coated pits for endocytosis and rapidly recycled back to the plasma membrane [319]. However, after the chronic stimulation of receptors, the internalized receptor is degraded in the lysosome and new receptors need to be synthesized [320]. 

#### 4.2.3. Immunomodulation and Inflammation

CGRP has been shown to play a role in pain transmission (e.g., migraine; for a comprehensive review on this topic, the reader is referred to Spekker et al. [321]) and has both pro- and anti-inflammatory activities: CGRP causes vasodilation, which promotes inflammation, and it increases cAMP production, which inhibits the release of inflammatory mediators [322,323,324]. CGRP can also modulate the differentiation, proliferation, and activities of immune cells, such as lymphocytes, cDCs, and macrophages, through various cytokines [325,326,327,328,329,330,331,332].

#### 4.2.4. Role of CGRP in the Cornea

CGRP-positive neurons in the trigeminal ganglia and corneal nerve fibers expressing CGRP are significantly more abundant than those positive for SP [25]. Similar to SP, CGRP is an important mediator in the nociceptive functions of corneal nerves and plays a role in the “trophic” efferent function of corneal sensory nerves [333,334]. Several in vivo studies have shown that CGRP plays an important role in corneal epithelial wound healing by facilitating corneal epithelial cell migration and differentiation [335]. During corneal epithelial wound healing, the CGRP-positive nerve fibers regenerate, and the concentration of CGRP increases in tears [336,337,338]. The level of CGRP in tears is also directly correlated with the lacrimal function [339]. Moreover, exogenous CGRP-treated corneas have a higher epithelial wound healing rate compared with control corneas [337]. These effects could be due to binding CGRP to corneal epithelial cells, resulting in the synthesis of chemotactic proteins, such as IL-8, and leukocyte infiltration, which can be inhibited by the CGRP receptor antagonist CGRP8–37 [179]. 

### 4.3. Adrenomedullin (AM)

AM belongs to the amylin/intermedin/CGRP family of polypeptides and was originally isolated from human pheochromocytoma in 1993 [84,340]. AM is widely distributed in numerous tissues and organs with a local paracrine and autocrine role in regulating various functions, such as vasodilatation, cell growth, hormone secretion, natriuresis, and antimicrobial effects [341,342,343].

#### 4.3.1. Transcriptional Regulation

**Figure 4:**
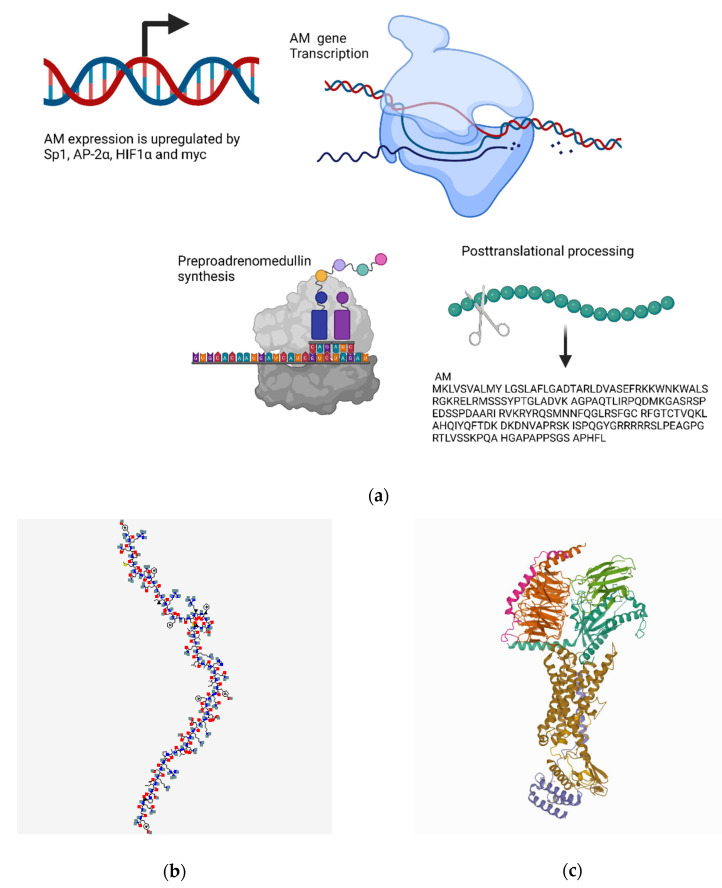
Neuropeptide AM and receptors: (**a**) transcription and synthesis of AM (created with BioRender.com accessed on 22 October 2021); (**b**) 2D structure image of AM (https://pubchem.ncbi.nlm.nih.gov/compound/56841671#section=2D-Structure, accessed on 22 October 2021); (**c**) CryoEM structure of the active adrenomedullin 1 receptor G protein complex with adrenomedullin peptide. Image from the RCSB PDB (rcsb.org) of PDB ID 6UUN [85].

AM is encoded by the AM gene contained in human chromosome 11 and in mouse chromosome 7 [344]. The transcription of the adrenomedullin gene can be synergistically induced by the actions of stimulatory protein 1 (Sp1) and AP-2α, which each bind to nonoverlapping sites within the promoter region [345]. Other important mediators of adrenomedullin transcription include the hypoxia-inducible factors (HIFs) and inflammatory cytokines. HIF-1α induces adrenomedullin expression in response to both hypoxia and IL-1β [346]. The myc transcription factor also regulates adrenomedullin transcription; however, myc has been shown to have different regulatory roles in different species. For instance, in mouse fibroblasts, myc is a potent repressor of adrenomedullin transcription [347]; however, both the rat and human adrenomedullin genes are transactivated by myc [348] (Figure 4a).

#### 4.3.2. Metabolism and Signaling

AM is a circulating peptide mostly found in biological fluids such as plasma (bound to complement factor H), urine, saliva, sweat, milk, amniotic fluid, and cerebrospinal fluid [349]. AM has a half-life of 16–20 min and is rapidly degraded by matrix metalloprotease 2 and aminopeptidase [341,350].

Three signal transduction pathways are activated by AM: the cAMP, Akt, and MAPK/ERK pathways [351,352,353]. AM activates adenylate cyclase and increases intracellular levels of cAMP, which causes PKA activation and increased calcium (Ca^2+^) efflux. Calcium release can also be stimulated by AM through phospholipase C activation and inositol-1,4,5-P3 formation [354]. However, the regulation of Ca^2+^ efflux may vary depending on the cell type and environment. The intracellular Ca^2+^ increase due to AM also causes the activation of the NO-dependent pathway, which inhibits endothelial cell apoptosis [355,356]. AM also activates the PI3K/Akt and MAPK/ERK signaling pathways in vascular endothelial cells and myocytes to promote endothelial cell growth, inducing cardioprotection and antiapoptotic effects [357,358,359].

#### 4.3.3. Immunomodulation and Inflammation

AM synergizes with stem cell factor and FMS-like tyrosine kinase-3 (Flt-3) ligand to induce the proliferation of primitive human CD34^+^CD38^−^lin^−^ cells and promotes the expansion of CD34^+^ progenitors in culture [360,361]. AM may be used to improve the expansion of hematopoietic stem cells from cord blood, which are of great importance for tissue engineering and clinical use [341,344].

#### 4.3.4. Role of Adrenomedullin in the Cornea

AM, along with its receptor complex CLR/RAMP2 expression (mRNA and protein), is more prevalent in the corneal epithelium versus the stroma plus endothelium of the naive cornea [362]. The expression is significantly increased after inflammation induced by thermal cautery, intrastromal suture placement, or ciliary nerve axotomy [362]. Although the role of AM/CLR/RAMP2 in the cornea is not very well understood, the sum length of suture-induced heme- and lymph-angiogenesis is reduced by the depletion of AM with siRNA compared with control siRNA, indicating that the modulation of AM in the cornea can reduce pathological corneal angiogenesis [363]. Furthermore, the ubiquitous temporal deletion of the CLR receptor by an inducible Cre-loxP system rapidly develops dilated corneoscleral lymphatics associated with corneal edema and inflammation [187]. Collectively, these studies indicate that AM may serve as a target for corneal angiogenesis. 

### 4.4. Vasoactive Intestinal Polypeptide (VIP)

VIP is a member of the secretin/glucagon superfamily, which includes secretin, growth hormone-releasing peptide (GHRP), and pituitary adenylate cyclase-activating peptide (PACAP) [364]. VIP is produced by neurons, endocrine, and immune cells, and it is known to function as an inhibitory neurotransmitter in both the central and peripheral nervous systems [364,365].

#### 4.4.1. Transcriptional Regulation

**Figure 5:**
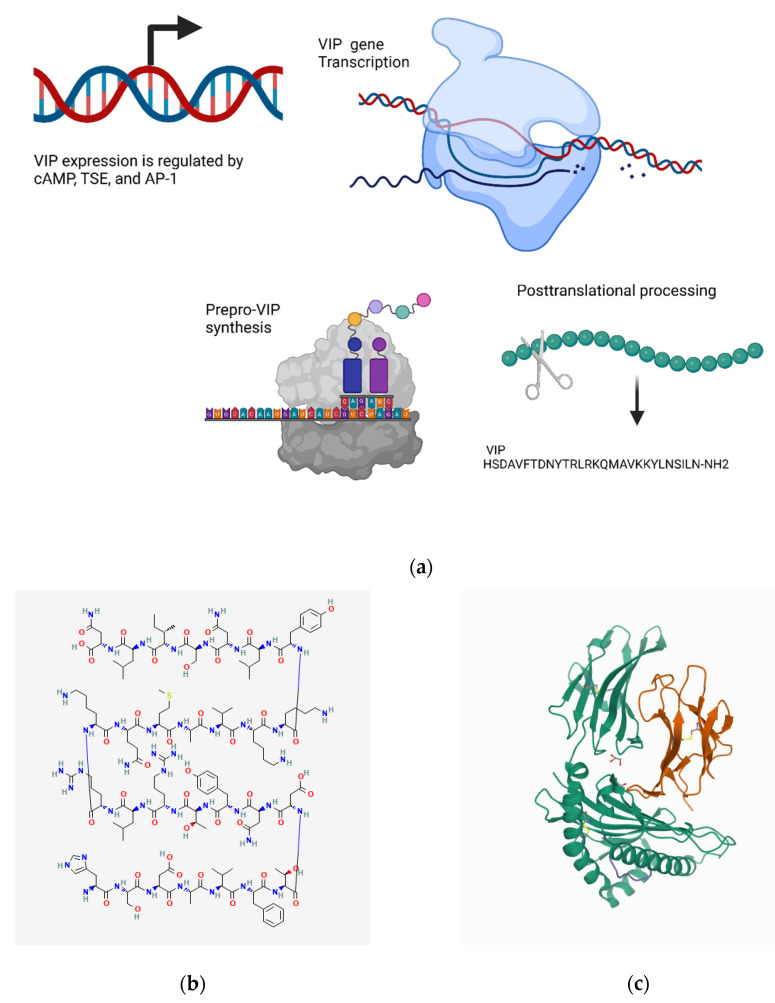
Neuropeptide VIP: (**a**) transcription and synthesis of VIP (created with BioRender.com accessed on 22 October 2021); (**b**) 2D structure image of VIP (https://pubchem.ncbi.nlm.nih.gov/compound/53314964#section=2D-Structure, accessed on 22 October 2021); (**c**) crystal structure of B*27:06 bound to the pVIPR peptide. Image from the RCSB PDB (rcsb.org) of PDB ID 5DEG [368].

The VIP gene contains multiple regulatory elements. For instance, in the immediate upstream of the VIP promoter, there is a cAMP-responsive element (CRE), and further upstream, a tissue-specifier element (TSE) has also been identified [366]. The CRE site is absolutely required for the cAMP-induction of VIP, as determined by the deletion of this element. Additionally, the deletion of the CRE site reduces the constitutive expression of VIP, whereas the deletion of the TSE site alone is sufficient for the silencing of constitutive expression [366]. A key transcription factor in regulating VIP gene expression is activator protein 1 (AP-1), a heterodimer of c-Fos and c-Jun (and related proteins). Evidence suggests that constitutive and inducible gene expressions are regulated, at least in part, by different AP-1 complexes [366]. Further control over VIP expression is achieved by enhancer/repressor elements, approximately 5 kb upstream of the promoter region [367] (Figure 5a).

#### 4.4.2. Metabolism and Signaling

The binding of VIP to its receptors causes an increase in the levels of cAMP, adenylate cyclase, and phospholipase C, thus initiating a downstream signaling cascade [369]. The VIP-bound receptors are internalized and then recycled to the cell membrane [370]. Therefore, a lag phase in the cellular response to VIP occurs when the receptors are saturated.

The effects of VIP are mostly produced either by cAMP-dependent or -independent pathways [371]. The cAMP-dependent pathway reduces the activity of nuclear factor κB (NFκB) via the phosphorylation of CREB (cAMP response element binding protein) by PKA leads to the binding of CREB to CREB-binding protein (CBP), which reduces its interaction with NFκB, or via the phosphorylation of MAP/ERK kinase (MEK) kinase 1 (MEKK1) by PKA, inhibiting the phosphorylation of the TATA-box binding protein (TBP) and reducing its affinity for NFκB and DNA [371,372]. The cAMP-dependent pathway also inhibits the phosphorylation of the Janus kinase/signal transducer and the activator of its transcription (JAK/STAT) pathway [373,374]. The cAMP-independent pathway inhibits the activity of inhibitory κB kinase (IκK), which prevents the nuclear translocation of NFκB subunits by increasing the stabilization of IκB/NFκB complexes [371,375].

#### 4.4.3. Immunomodulation and Inflammation

VIP has been shown to have both pro- and anti-inflammatory effects through the modulation of immune cells. Depending on the timepoint or receptor type, VIP may have different effects on developing cDCs, inducing an inhibitory or immunogenic/mature cDC phenotype [376,377,378]. VIP primes the oxidative response of neutrophils to formyl-methionyl-leucyl-phenylalanine (FMLP) and phorbol myristate acetate (PMA) [379,380]. VIP has autocrine functions in mast cells that produce VIP and histamine through the classical IgE-mediated pathway, and VIP can also stimulate the release of histamine by mast cells, leading to inflammatory effects [381,382].

Studies show that VIP can also have an anti-inflammatory effect by inhibiting the lipopolysaccharide (LPS)- or interferon (IFN)-γ-induced synthesis of cytokines TNF-α, IL-6, IL-12, and nitric oxide (NO) by macrophages and monocytes via the cAMP-dependent pathway (JAK/STAT) [364,383,384]. In allergic or parasitic diseases, the increased innervation of VIP-positive nerves is associated with eosinophil accumulation and the inhibition of IL-16 synthesis, as well as the chemotaxis of immune cells [67,385]. Besides the inhibition of inflammatory factors, VIP also stimulates the production of anti-inflammatory cytokines such as IL-10 [386,387].

#### 4.4.4. Role of VIP in the Cornea

Studies have revealed the role of VIP in exerting anti-inflammatory effects and modulating wound healing in alkali-burned corneas, microbial keratitis, and corneal allograft survival [388,389,390]. VIP exerts these corneal effects in a sonic hedgehog (SHH)-dependent manner, which is an important downstream signaling molecule of the VIP/VPAC1 pathway [391]. Blocking VIP-VPAC1 signaling in corneas delays healing in normal mouse corneas, and the addition of exogenous VIP improves corneal wound healing in diabetic mice [392]. In addition, VIP also promotes corneal nerve regeneration by inducing the expression of neurotrophic factors NGF and CNTF [338].

VIP also promotes the survival of corneal endothelial cells under oxidative stress and, therefore, improves the integrity of corneal endothelial cells during donor cornea tissue storage [393,394,395]. Moreover, VIP significantly accelerates corneal epithelial wound closure in a murine model of diabetes [391]. VIP also improves corneal transplantation outcomes by limiting inflammatory cytokine (IFN-γ, TNF-α)-mediated apoptosis, thus increasing endothelial cell density and corneal graft survival [396].

VIP has a well-established role in improving outcomes in models of bacterial and fungal keratitis. Its benefit in these settings may be due to several mechanisms, including: the restoration of the extracellular matrix [397], the modulation of pro- and anti-inflammatory Toll-like receptors [398], and the downregulation of adhesion molecules [399]. In fungal keratitis, VIP treatment downregulates pro-inflammatory cytokine expression, and this effect can be reversed by a VIP antagonist [400].

### 4.5. Pituitary Adenylate Cyclase-Activating Polypeptide (PACAP)

PACAP belongs to the VIP/glucagon/secretin family, with a well-conserved amino acid sequence sharing 68% homology with VIP [364]. PACAP is involved in various developmental and physiological processes, such as neural differentiation, neurite outgrowth, neuroprotection, neurotransmission, hormone secretion, vasodilation, and immunosuppression [96,401,402,403,404,405,406,407,408].

#### 4.5.1. Transcriptional Regulation

**Figure 6:**
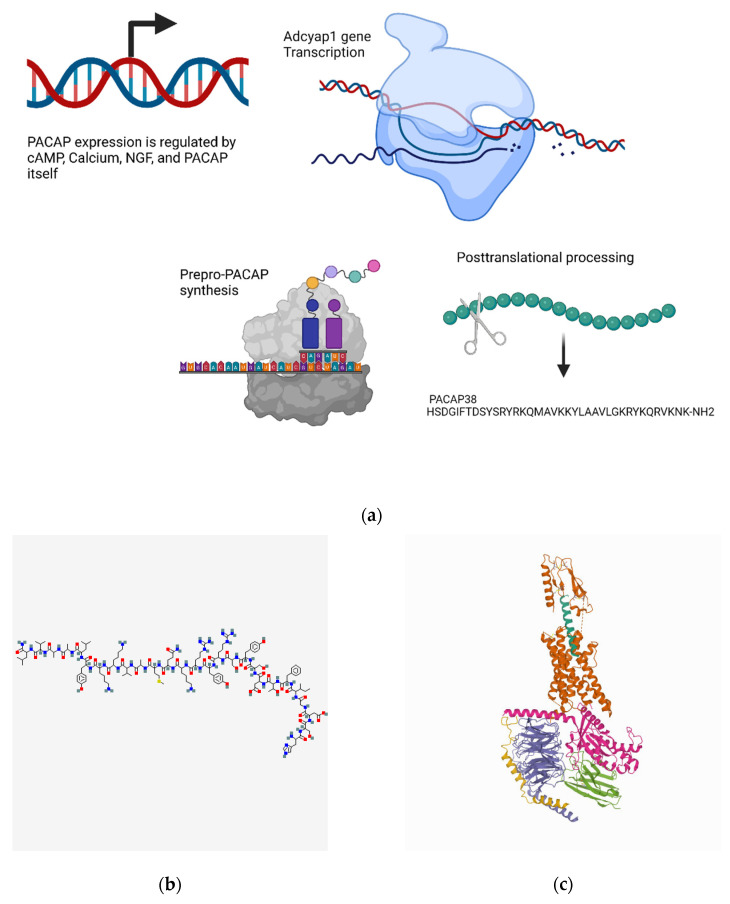
Neuropeptide PACAP and PAC1R: (**a**) transcription and synthesis of PACAP (created with BioRender.com accessed on 22 October 2021); (**b**) 2D structure image of PACAP (https://pubchem.ncbi.nlm.nih.gov/compound/137699541#section=2D-Structure, accessed on 22 October 2021); (**c**) Cryo-EM structure of the human PAC1 receptor coupled to an engineered heterotrimeric G protein. Image from the RCSB PDB (rcsb.org) of PDB ID 6LPB [412].

The PACAP gene, *Adcyap1*, also contains a CRE site, and cAMP and Ca^2+^ act in a synergistic manner to upregulate PACAP transcription [409]. Interestingly, PACAP also appears capable of inducing its own gene expression, and this occurs in a protein kinase C-dependent manner. Furthermore, PACAP and NGF can act synergistically to promote PACAP transcription, and this appears to involve extracellular signal-regulated kinase (ERK) signaling [410]. Novel splice variants, mostly within the 5’UTR region of the PACAP transcript have been identified, and their expression occurs in a tissue-specific manner [411]. One splice variant within the coding region of the transcript, although distinct from the PACAP region, has been identified in activated T cells [411]. The functional significance of these variants remains unclear, although they may regulate the translation of PACAP in a tissue-specific manner (Figure 6a).

#### 4.5.2. Metabolism and Signaling

VPAC1 and VPAC2 receptors primarily activate the adenylate cyclase pathway, whereas PAC1-R activates both adenylate cyclase and phospholipase C [413]. PACAP/PAC1-R binding is associated with the recruitment of Gαs and Gαq/11, activating plasma membrane adenylyl cyclase, which increases cellular cAMP and initiates PKC/phospholipase C downstream signaling [103,414,415,416]. After PACAP/PAC1-R internalization and endosomal signaling, a MAPK and Akt signaling cascade can be initiated [416,417,418,419,420]. The internalized vesicles are rapidly colocalized with β-arrestin, and the endosomal markers Rab5 or Rab7a suggest vesicular trafficking to lysosomal compartments for potential degradation [421,422,423,424,425]. PACAP promotes JunB and inhibits c-Jun phosphorylation via the MEKK1/MEK4/JNK pathway, and it inhibits TBP phosphorylation through the MEKK1/MEK3/6/p38 MAPK pathway, resulting in the transcriptional inactivation of various cytokine promoters [426,427,428]. 

#### 4.5.3. Immunomodulation and Inflammation

PACAP has both pro- and anti-inflammatory roles through the modulation of innate and acquired immunity, depending on the physiological and pathological conditions [429,430]. In LPS-induced macrophages, PACAP inhibits the secretion of several pro-inflammatory mediators, such as TNF-α, IL-12, IL-1, IL-6, and nitric oxide (NO) via both the cAMP-dependent and cAMP-independent pathways, whereas the production of the anti-inflammatory cytokine IL-10 is stimulated via the cAMP-dependent pathway [431,432]. PACAP-treated macrophages can induce Th2-type cytokines (IL-4 and IL-5) and inhibit Th1-type cytokines (IFN-γ, IL-2) in Ag-primed CD4 T cells [433,434]. PACAP also plays a role in thymic T cell maturation, inhibiting the induced cell death of T lymphocytes from glucocorticoid-induced apoptosis [430,435].

#### 4.5.4. Role of PACAP in the Cornea

PACAP expression has been reported in corneal nerve fibers and small-to-medium-sized neurons in the trigeminal ganglion via immunocytochemistry [195]. PACAP plays a role in tear secretion, and, therefore, PACAP-knockout mice have been shown to develop dry eye-like symptoms such as corneal keratinization and reduced tear production [190]. PACAP eyedrops can stimulate tear secretion via the AC/cAMP/PKA pathway by stimulating the translocation of aquaporin 5 from the cytosol to the membrane of lacrimal acinar cells [190]. Exogenous PACAP application can also induce corneal nerve regeneration, improve corneal sensitivity, and accelerate corneal epithelial wound healing after injury or refractive surgery [70,436,437].

Corneal endothelial cells also express PACAP and all three receptors. PACAP has been shown to have a protective role in corneal endothelial cells against ultraviolet B exposure by increasing the tight junction protein expression and transepithelial electrical resistance [70,438]. PACAP also protects against growth factor deprivation-induced decreases in corneal endothelial cell viability by inducing epidermal growth factor receptor phosphorylation and MAPK/ERK1/2 pathway activation [439].

### 4.6. Neuropeptide Y (NPY)

NPY belongs to the neuroendocrine polypeptide NPY family, which also includes peptide YY (PYY) and pancreatic polypeptide [440]. It was first isolated from the porcine hypothalamus in 1982 [441]. NPY is co-stored and co-released with norepinephrine in the peripheral postganglionic sympathetic nerve in peripheral tissues, such as the retina, smooth muscle, the intestine, bone marrow, and the thymus [442]. Studies have also shown that stimulated or mature immune cells can also synthesize NPY [443,444,445].

#### 4.6.1. Transcriptional Regulation

**Figure 7:**
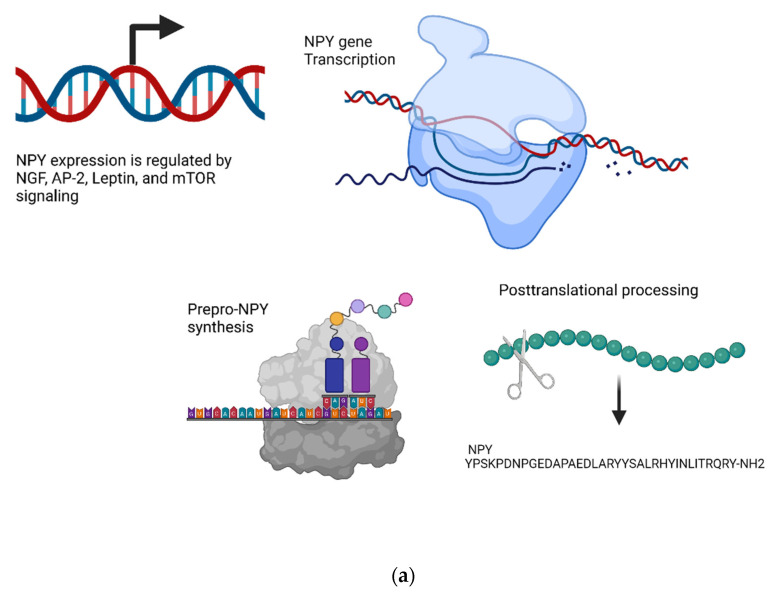

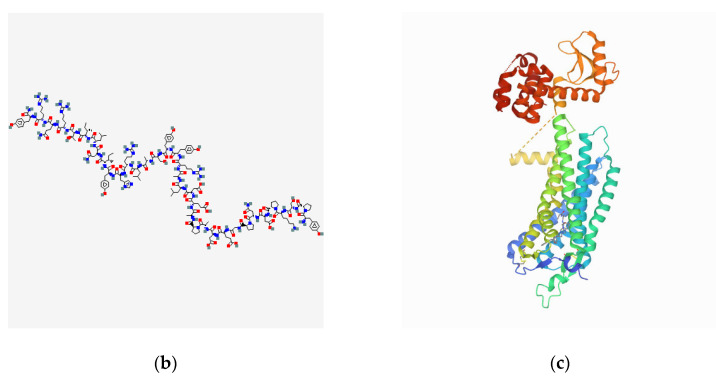
Neuropeptide Y and receptor: (**a**) transcription and synthesis of NPY (created with BioRender.com accessed on 22 October 2021); (**b**) 2D structure image of NPY (https://pubchem.ncbi.nlm.nih.gov/compound/16132350#section=2D-Structure, accessed on 22 October 2021); (**c**) the crystal structure of a human neuropeptide Y Y1 receptor with UR-MK299. Image from the RCSB PDB (rcsb.org) of PDB ID 5ZBQ [451].

Similar to the other transcriptional mechanisms discussed so far, NGF induces an upregulation in NPY transcription, and there is a synergistic effect between combinatorial treatment with NGF and the activators of cAMP and protein kinase C [446]. Further characterization of the NPY gene has identified an AP-2 binding site near the promoter region, and this represents one mechanism by which NGF can induce transcription [447]. NPY promotes feeding behavior, so, not surprisingly, the satiety hormone leptin regulates NPY transcription. However, this process is more complex than initially thought, as leptin may upregulate or downregulate NPY transcription, and the effect may be dictated in a cell type-specific manner. The leptin-mediated downregulation of NPY appears to be mediated through SOCS3, which has a putative binding site within the NPY promoter region and serves as a negative regulator of transcription within the hypothalamus [448]. However, in vitro studies using various neural cell lines have demonstrated that leptin induces an upregulation in NPY transcription and is dependent on JAK-STAT signaling; importantly, however, no SOCS3 expression was detected in these cell lines [448,449]. Another negative regulator of NPY transcription appears to be a mammalian target of rapamycin (mTOR) signaling. The inhibition of mTOR signaling by rapamycin leads to a robust increase in NPY transcription, and a similar effect has been seen in treatments with dexamethasone. Notably, there was no synergistic effect found between dexamethasone and rapamycin treatment, suggesting that the upregulation of NPY by dexamethasone is mediated by mTOR inhibition [450] (Figure 7a).

#### 4.6.2. Metabolism and Signaling

NPY receptors mediate the inhibition of cAMP synthesis, the activation of phospholipase C, and the mobilization of intracellular Ca^2+^ [452,453]. NPY/Y1 receptor is desensitized by rapid, clathrin-dependent internalization and recycled at the plasma membrane via sorting/early endosomes (SE/EE) and recycling endosomes (RE) [454].

NPY exerts its effects primarily through Y1 receptors activating Ca^2+^-dependent pathways: PKC and calcium/calmodulin-dependent kinase II (CaMKII) [455,456,457]. These pathways are also amplified by Y5 the receptor-mediated, Ca^2+^-independent inhibition of the AC/PKA pathway at the high-affinity peak, leading to an ERK1/2 signaling cascade [458].

#### 4.6.3. Immunomodulation and Inflammation 

Various studies have reported the close proximity and interaction between NPY-positive nerves and immune cells [459]. NPY is expressed by the sympathetic nervous system and immune cells, and it is upregulated under inflammation. NPY modulates immune cell function in a paracrine or autocrine manner [460,461]. NPY/Y1R interaction also has both pro- and anti-inflammatory effects on immune cells.

NPY has a promigratory effect on cDCs, which leads to increased inflammation; however, the maturation of cDCs and the synthesis of inflammatory cytokines are inhibited in a murine model of inflammation [462]. NPY also upregulates the expression of IL-6 and IL-10 via human immature cDCs [463,464]. NPY can also enhance opsonin-dependent phagocytosis via human neutrophils, and cDCs in Y1R-knockout mice have impaired phagocytic capacity, hindering T cell activation [465,466]. NPY also modulates the recruitment and chemotaxis of lymphocytes by affecting their adhesion and tropism, depending on the type of receptor, tissue, and age [462,467]. NPY also inhibits the proliferation of lymphocytes, but this effect declines with aging [468]. NPY/Y1R interaction in bone marrow decreases the number of pro-B, pre-B, and immature B cells and increases that of mature B cells [469]. NPY/Y1R binding can also regulate the recruitment of monocytes and macrophages in rodents by decreasing their adhesion and promoting migration [470].

NPY can also exert pro-inflammatory effects on macrophages by promoting the synthesis of pro-inflammatory cytokines [462]. NPY significantly increases the expression of TNF-α, C-reactive protein, and monocyte chemoattractant protein 1 (MCP1) in macrophages during inflammation [471]. However, NPY also exerts anti-inflammatory effects by stimulating the release of macrophage anti-inflammatory cytokines IL-10 and IL-1RA and transforming macrophages into the M2-like phenotype [445].

#### 4.6.4. Role of NPY in the Cornea

NPY has been shown to be distributed in the human corneal epithelium, corneal myofibroblasts, and corneal nerves near the limbus, as identified by immunohistochemistry [472]. Corneal epithelial cells and myofibroblasts also express the NPY receptor, suggesting an autocrine or paracrine role in corneal homeostasis and repair [472].

Interestingly, one study demonstrated that, in diabetic patients with ocular surface disease, NPY was significantly increased compared with healthy controls via conjunctival impression cytology [473]. The NPY levels also correlated with increases in ICAM-1, possibly indicating the role of NPY in the inflammation of the ocular surface [473]. However, the same study failed to detect an increase in NPY among allergic conjunctivitis patients [474]. NPY has also been reported to be closely involved with angiogenesis and wound healing in mouse corneas [475]. In that study, corneal micropockets were created with a modified von Graefe cataract knife in both eyes of C57BL/6 wt or NPY Y2−/− mice, and a micropellet of aluminum sulfate coated with slow-release polymer-hydron, containing FGF-2, VEGF, or NPY, was implanted into each corneal pocket to induce neovascularization in the corneal avascular tissue [475]. The measured angiogenic responses, such as vessel length, clock hours, and neovascularization area, were all significantly greater in the NPY-implanted corneas than in the negative controls [475]. A selective ligand for the Y2 receptor induced a similar angiogenic pattern to unprocessed NPY in mouse corneas, suggesting that the Y2 receptor subtype is responsible for the mediation of NPY-stimulated angiogenesis [475]. Furthermore, the deletion of the Y2 receptor in mice impaired the angiogenic response in vivo, and NPY completely failed to induce corneal blood vessel growth in these knockout mice [475].

### 4.7. Somatostatin (SST)

SST is a cyclic peptide that belongs to the somatostatin family of regulatory peptides. SST mainly produces a neuroendocrine inhibitory effect and, hence, is also known as a growth hormone-inhibiting hormone [476]. SST is distributed throughout the nervous system, gastrointestinal tract, and pancreas. It is also known to regulate neurotransmission, memory formation, and anti-angiogenesis in addition to inhibiting endocrine and exocrine secretions [477].

#### 4.7.1. Transcriptional Regulation

**Figure 8:**
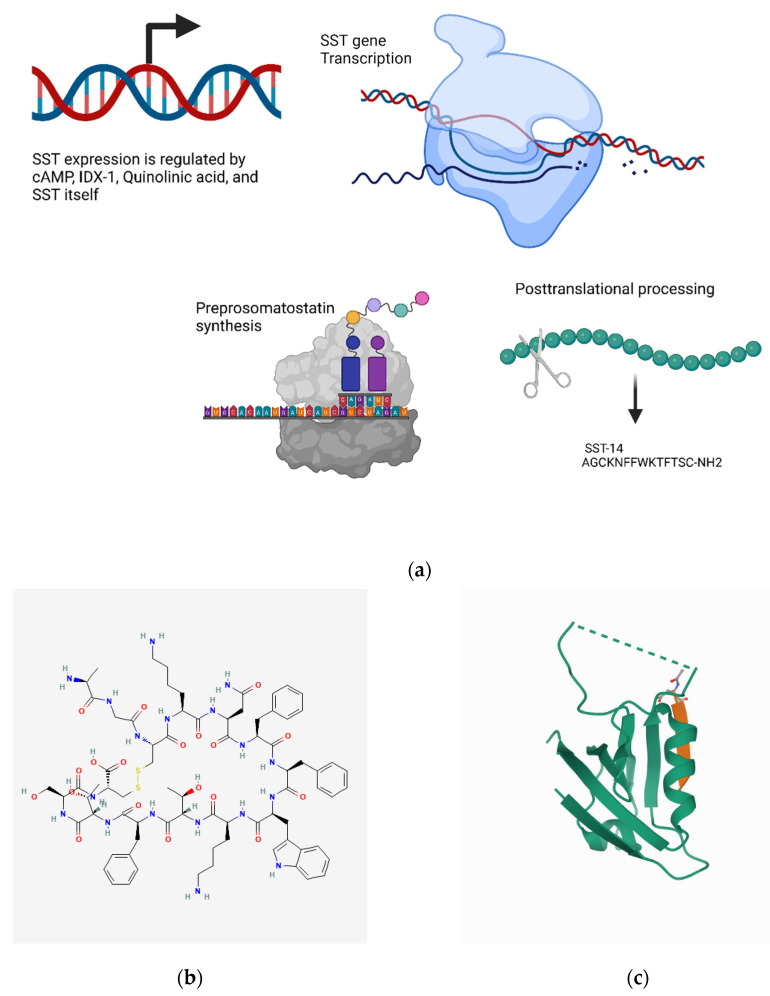
Neuropeptide SST and receptor: (**a**) transcription and synthesis of SST (created with BioRender.com accessed on 22 October 2021); (**b**) 2D structure image of SST (https://pubchem.ncbi.nlm.nih.gov/compound/16129706#section=2D-Structure, accessed on 22 October 2021); (**c**) PDZ domain from rat Shank3 bound to the C terminus of somatostatin receptor subtype 2. Image from the RCSB PDB (rcsb.org) of PDB ID 6EXJ [482].

The somatostatin gene contains three sites upstream of its promoter to which IDX-1, a homeobox transcription factor, binds. This binding increases the transcription of somatostatin, and the site-directed mutagenesis of these binding sites abrogates the effect [478]. Quinolinic acid (an NMDA receptor agonist) and NMDA itself have been shown to induce somatostatin transcription; pre-treating with an NMDA antagonist blocks the induction of somatostatin transcription [479]. However, the exact signaling mechanisms linking the NMDA receptor to the transcription of somatostatin are not clear [479]. Additionally, CRE sites are present upstream of the somatostatin promoter, and, as expected, cAMP induces the transcription of the somatostatin gene [480]. Interestingly, there is evidence to suggest that this CRE site functions as an enhancer of basal somatostatin transcription, even in the absence of cAMP [481] (Figure 8a).

#### 4.7.2. Metabolism and Signaling

SST has a plasma half-life between 1 and 3 min due to proteolytic degradation. SST interaction with different SST receptor subtypes mediates various signal transduction pathways depending on the cell type, including adenylate and guanylate cyclase; phospholipase A2 and C; K^+^ and Ca^2+^ channels; Na^+^–H^+^ exchanger; Src; Erk1/2; p38 MAPK; and tyrosine phosphatases [483,484,485,486]. SSTR2 and SSTR5 can modulate growth hormone, insulin, and glucagon release; SSTR3 can induce apoptosis; and SSTR1, SSTR2, and SSTR5 can inhibit the cell cycle [487].

#### 4.7.3. Immunomodulation and Inflammation

Studies have shown SSR expression in immune cells using both fluorescent and radiolabeled SST, and the SSR expression is correlated with the activation and/or proliferation state of the immune cells [488,489]. SST is expressed in the nerves innervating the lymphoid organs and can modulate the responses of lymphocytes by influencing adhesion and motility [490,491,492]. SST can reduce the phagocytosis of human monocytes and macrophages. SST can both suppress and stimulate T lymphocyte proliferation and has antiangiogenic properties [493,494,495]. SST also modulates the immunoglobulin (IgE and IgG) production of plasma B cells [496].

#### 4.7.4. Role of SST in the Cornea

SST has been detected in tear fluid, and the main source of SST in the ocular surface seems to be the lacrimal gland [201]. The expression of SST receptors has been reported in different tissues of the ocular surface, including the cornea, which expresses SSTR1 and SSTR2 [201,497]. This suggests the autocrine and paracrine role of SST in corneal immunology [201]. SST delivered in pellets containing 90 ng of basic fibroblast growth factor inhibited corneal neovascularization in a rat corneal pocket model of induced neovascularization [498]. In that study, a 200 ng dose of SST showed a significant inhibition of both the length and area of corneal neovascularization on day 7 [498]. SST (10 ng/μL) significantly promotes the healing of corneal defects in vivo in an alkali-induced corneal injury mouse model, but the mechanism remains elusive, as SST does not enhance the proliferation and migration of the human corneal epithelial cell line in vitro [499].

### 4.8. α-Melanocyte Stimulating Hormone (α-MSH)

α-MSH belongs to the melanocortin family of peptides, which includes α-, β-, γ-melanocyte-stimulating hormone (MSH) and adrenocorticotropic hormone (ACTH) [120,500]. Melanocortin peptides play an important role in modulating host defense mechanisms in mammals [124]. 

#### 4.8.1. Transcriptional Regulation

**Figure 9:**
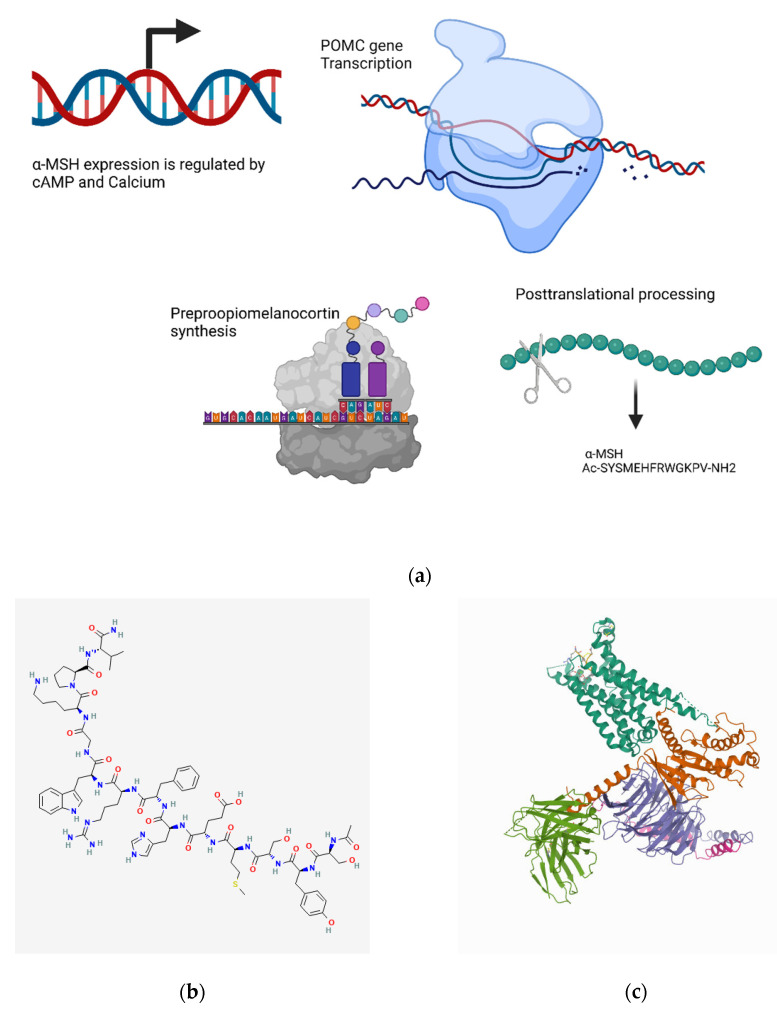
Neuropeptide α-MSH and MC4R: (**a**) transcription and synthesis of α-MSH (created with BioRender.com accessed on 22 October 2021); (**b**) 2D structure image of α-MSH (https://pubchem.ncbi.nlm.nih.gov/compound/44273719#section=2D-Structure, accessed on 22 October 2021); (**c**) melanocortin receptor 4 (MC4R) Gs protein complex. Image from the RCSB PDB (rcsb.org) of PDB ID 7AUE [504].

α-MSH is encoded by the proopiomelanocortin hormone (POMC) gene [501]. Cells that can synthesize POMC, such as macrophages, keratinocytes, and neurons, produce α-MSH [120]. The release of corticotropin-releasing hormone due to infection or stress stimulates the production of POMC, which is ultimately processed into α-MSH peptides [502]. Both cAMP and intracellular Ca^2+^ enhance the expression of the POMC gene, although the POMC promoter lacks CRE sites [503] (Figure 9a).

#### 4.8.2. Metabolism and Signaling

POMC is cleaved by prohormone convertases (C-terminal basic amino acids removed by carboxypeptidase E enzyme), amidated by peptidyl α-amidating monooxygenase enzyme, and acetylated by N-acetyl-transferase enzyme to form active acetyl α-MSH [120,501]. The removal of valine residue at the C-terminal catalyzed by prolylcarboxypeptidase inactivates α-MSH [120,505]. 

#### 4.8.3. Immunomodulation and Inflammation

α-MSH can suppress both innate immune-mediated and adaptive immune-mediated inflammation [124,506,507,508]. α-MSH suppresses the production of inflammatory cytokines, ROIs, and NO in macrophages induced by endotoxin, IL-1β, and TNFα [502,509,510]. In addition, α-MSH activates suppressor cell activity in macrophages and suppresses the chemotactic activity of macrophages and neutrophils [69,511]. α-MSH exerts immunomodulatory effects on antigen-presenting cells, thereby promoting the expansion of inducible regulatory T cells [512]. Thus, α-MSH suppresses T cell-mediated inflammation by regulating effector T cell functions and suppressing the production of IFN-γ [508,513]. The immunomodulatory and anti-inflammatory effects of α-MSH (through the inhibition of NFκB activation), the blockade of accessory signals, and the induction of suppressor factors could be an important pathway driving immune tolerance [121,507,510].

#### 4.8.4. Role of α-MSH in the Cornea

The cornea is an immune-privileged tissue, and melanocortin pathways have been shown to be important in the suppression of pro-inflammatory signals, the regulation of the immune response, and in the induction of tolerance on the ocular surface [509,514]. A-MSH also induces the production of TGF-β2, which is a major immunoregulatory molecule in the aqueous humor [514,515,516]. It has also been observed that α-MSH expression within the eye declines in the setting of intraocular autoimmune disease; however, the regulators of α-MSH expression remain unclear [514,517]. Moreover, in an experimental autoimmune uveitis model, mice treated with α-MSH had a reduction in inflammation, underscoring the immunoregulatory function of α-MSH within the eye [517,518].

Furthermore, α-MSH has well-established roles in several ocular surface diseases. The local delivery of α-MSH in a corneal transplantation model resulted in decreased IFN-γ and IL-2 gene expression and improved graft survival compared to controls [519]. Additional work in this area has further revealed that α-MSH enhances corneal endothelial cell survival, likely mediated through MC1R as a knockdown of this receptor’s decreased graft survival [520]. Recently, it has been shown that α-MSH reduces the severity of *Aspergillus fumigatus* keratitis [521]. In a scopolamine-induced model of dry eye disease, α-MSH promoted tear secretion and survival and restored goblet cell function [522,523].

### 4.9. Galanin (GAL)

Galanin belongs to the galanin family of peptides, including galanin-message-associated peptide (GMAP), galanin-like peptide (GALP), and alarin [524]. GAL was discovered at the Karolinska Institute in Stockholm in the 1980s [441].

#### 4.9.1. Transcriptional Regulation

**Figure 10:**
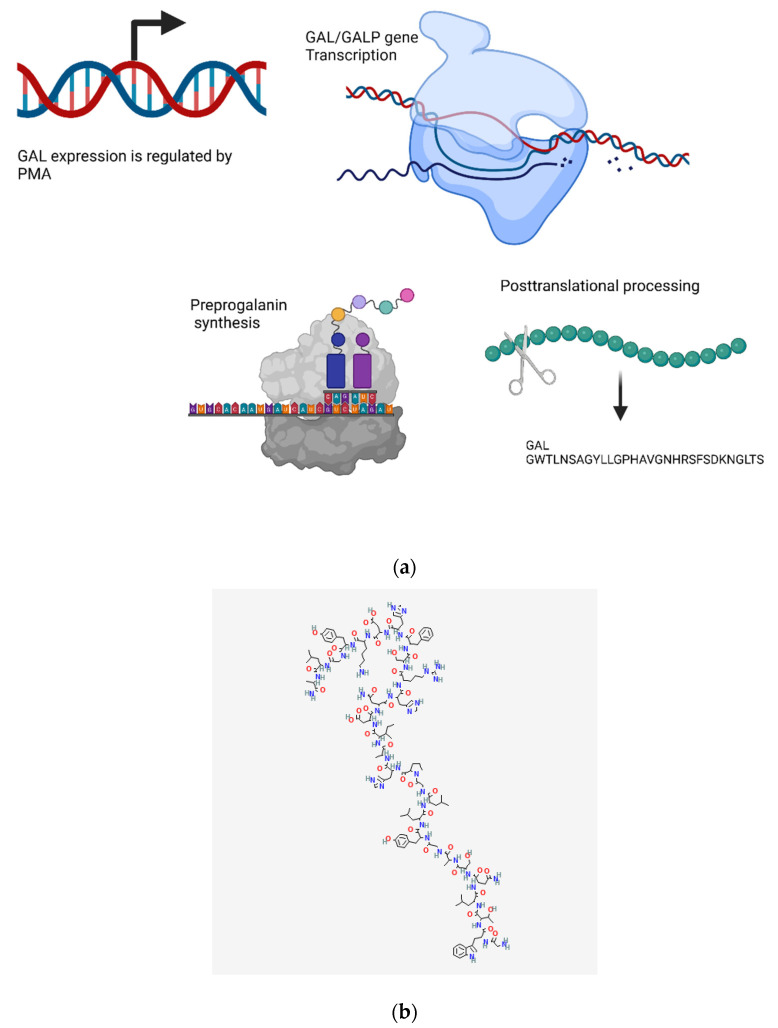
Neuropeptide GAL: (**a**) transcription and synthesis of GAL (created with BioRender.com accessed on 4 November 2021); (**b**) 2D structure image of GAL (swine) (https://pubchem.ncbi.nlm.nih.gov/compound/16174786#section=2D-Structure, accessed on 22 October 2021).

GAL is encoded by two separate genes: GAL/GMAP prepropeptide and galanin-like peptide (GALP) [525,526,527]. cJun/cFos proteins and the phorbol ester PMA can transactivate the GAL gene with a response mapped to the GAL promoter region where a CRE-like element binds to PMA, inducing gene expression mainly through the actions of Jun/ATF and CREB/ATF heterodimers [527,528] (Figure 10a). 

#### 4.9.2. Metabolism and Signaling

GAL exerts its effects via interaction with GAL receptors resulting in the activation of multiple transduction pathways. The GAL1 receptor interacts with the Gαi/αo class of G-proteins to inhibit adenylate cyclase (AC) and open GIRK channels [529,530,531]. GAL1 can also activate MAPK through a βγ-subunit of Gαi-mediated, PKC-independent mechanisms [532]. GAL2 can also stimulate MAPK activity through a PKC and the Gαo type of G-protein-dependent mechanisms [532]. Similarly, the activation of the Gαi/αo type of G-proteins by GAL2 can inhibit forskolin-stimulated cAMP production and inhibit CREB [531,533]. In addition, GAL2 can interact with the Gαq/11 class of G-proteins to stimulate phospholipase C activity and intracellular phosphoinositol turnover to release intracellular Ca^2+^ into the cytoplasm and open Ca^2+^-dependent channels [135,138,532]. Opening Ca^2+^ channels can phosphorylate PKB and suppress caspase-3 and caspase-9 activity [534,535]. GAL3 signaling is not very well understood, but studies have shown that GAL3 can activate the Gαi/αo type of G-proteins, resulting in AC inhibition and GIRK channels opening [138,536].

#### 4.9.3. Immunomodulation and Inflammation 

Galanin is widely expressed throughout the nervous and immune system, including in lymphoid organs, monocytes, macrophages, B cells, and T cells (both CD4^+^ and CD8^+^) [524]. GAL can affect these cells via the paracrine or endocrine signaling pathways. Since both PKA and PKC play a role in immunomodulation, the galanin system could be involved. During inflammation, GAL expression is markedly upregulated in the peripheral tissues, and various studies using animal models have reported the role of GAL in immunomodulation [524]. For example, GAL can modulate neutrophil sensitivity and IFNγ synthesis by NK cells when stimulated by the chemokines IL-12 and IL-18 [210,212,537]. In the nervous system, GAL is also suggested to be a modulator of inflammatory pain and nociception [538,539,540], with the inhibitory or excitatory effects depending on GAL concentration and the type of pain stimulus [541,542]. Studies using mouse models with GAL overexpression or knockout have shown that GAL affects the sensitivity of these animals to acute pain [543,544].

#### 4.9.4. Role of GAL in the Cornea

In the cornea, GAL receptors 1–3 have been detected by immunohistochemistry in the basal layers of the epithelium, stroma, and endothelium [545]. These receptors could have a role in angiogenesis or wound healing through interactions with GAL in the tear film since GAL expression has been reported in the lacrimal glands [546,547]. Further studies are necessary to confirm the role of these receptors.

### 4.10. Methionine Enkephalin (Met-Enkephalin, MENK, [Met5]Enkephalin) or Opioid Growth Factor (OGF)

Enkephalins were first isolated as endogenous opioids in brain extracts in 1975 [548]. The physiological effects of enkephalins include their role in cell division, migration, differentiation, viability, analgesia, angiogenesis, neuroprotection, and wound repair [500,549]. MENK is a naturally occurring opioid peptide that is a member of the endogenous opiate family. MENK is present throughout the nervous system and at low concentrations in blood [549]. 

#### 4.10.1. Transcriptional Regulation

**Figure 11:**
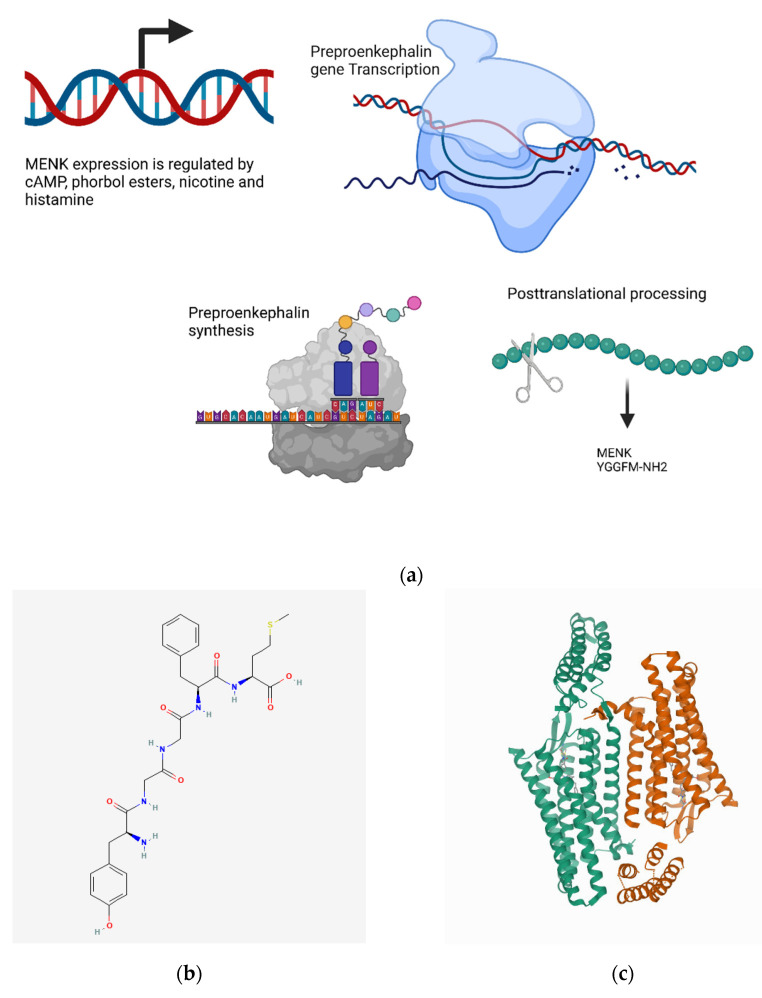
Neuropeptide MENK and opioid receptor: (**a**) transcription and synthesis of MENK (created with BioRender.com accessed on 22 October 2021); (**b**) 2D structure image of MENK (https://pubchem.ncbi.nlm.nih.gov/compound/443363#section=2D-Structure, accessed on 22 October 2021); (**c**) crystal structure of the active delta opioid receptor in the complex with the small molecule agonist DPI-287. Image from the RCSB PDB (rcsb.org) of PDB ID 6PT3 [553].

MENK is encoded by the preproenkephalin gene, which is composed of three exons that are separated by two introns, codes for six copies of [Met5]-enkephalin, and one copy of [Leu5]-enkephalin [139,550,551]. The expression of the preproenkephalin gene is regulated by cAMP, phorbol esters, nicotine, and histamine [552] (Figure 11a).

#### 4.10.2. Metabolism and Signaling

The biosynthesis of MENK involves the proteolysis and peptide cleavage of the proenkephalin prohormone within the secretory granules of the Golgi apparatus [554]. The biodegradation of enkephalins occurs via the cleavage of the Tyr-Gly bond via hydrolysis, followed by further degradation into shorter peptides (2–4 amino acids long) by nonspecific enkephalinases and aminopeptidases (NAP and NAP-2 in tissues; CD10 and CD13 in plasma) [555]. The half-life of enkephalins is 2–5 min in blood circulation [556,557].

The binding of enkephalins to opioid receptors dissociates the Gα and Gβγ subunits, which results in reducing K^+^ and Ca^2+^ influx in the cells. The Gα subunit hyperpolarizes the cell via direct interactions with inward-rectifying K+ channels and reduces the cAMP-dependent Ca^2+^ influx by inhibiting AC activity and cAMP formation. The Ca^2+^ influx is further reduced by the direct binding of the Gβγ subunit to the Ca^2+^ channels [558]. The resulting upregulation of p16 and p21 cyclin-dependent kinases halts the progression of the cell cycle from the G0/G1 phase to the S phase [559,560].

#### 4.10.3. Immunomodulation and Inflammation

Opioid receptors have been detected in the membranes of immune cells, including T cells, NK cells, macrophages, and dendritic cells. Studies have shown that MENK and/or its active metabolites have neuroendocrine functions in modulating pain sensitivity and immunomodulatory roles, including the upregulation of CD8^+^ T cell activity, the inhibition of Treg activity, the stimulation of macrophage phagocytosis, enhancing antigen processing capacity of DCs, the proliferation of CD4^+^ Th1 cells and B cells, and the stimulation of NK cell responses [549,561,562,563,564,565]. However, MENK seems to modulate the immune function only in the presence of a strong immunostimulatory signal [566]. 

#### 4.10.4. Role of MENK in the Cornea

MENK and its receptors have been shown to be present in the corneas of various species, including humans, mice, rats, and rabbits, and MENK is derived in an autocrine manner [158]. Studies using explant cultures and in vivo models of epithelial wound healing in rabbit corneas have demonstrated that MENK suppresses wound healing, and exposure to opioid antagonists, such as naloxone or naltrexone, blocks this effect [555,567,568]. Corneal wound healing is inhibited in the presence of MENK because it acts as a negative growth factor to repress cell division, DNA synthesis, and cell migration [567]. These effects are exerted via opioid receptor signaling as they can be blocked by disrupting opioid–receptor interactions using opioid antagonists [569].

### 4.11. Neurotensin (NT)

NT belongs to the neurotensin family of peptides, with various neuromodulatory effects on both the central and peripheral nervous systems [570]. NT was isolated as an endogenous tridecapeptide from bovine hypothalamic extracts, and the first observed property of NT was vasodilation [571]. NT can act both as a neurotransmitter and as a hormone in the body, regulating energy balance and control over homeostasis.

#### 4.11.1. Transcriptional Regulation

**Figure 12:**
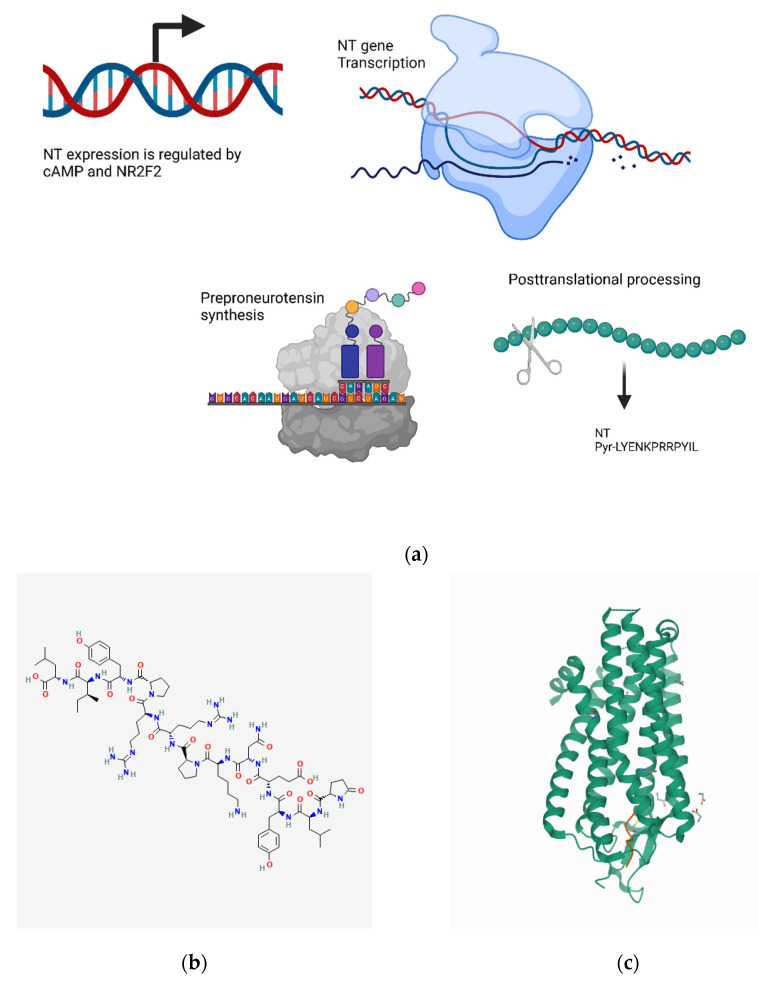
Neuropeptide NT and NTS1 receptor: (**a**) transcription and synthesis of NT (created with BioRender.com accessed on 22 October 2021); (**b**) 2D structure image of NT (https://pubchem.ncbi.nlm.nih.gov/compound/25077406#section=2D-Structure, accessed on 22 October 2021); (**c**) high-resolution structure of thermostable agonist-bound neurotensin receptor 1 mutant without lysozyme fusion. Image from the RCSB PDB (rcsb.org) of PDB ID 4BUO [576].

NT is encoded by the neurotensin gene, which encodes a common precursor for two peptides: neuromedin N and NT [572]. The constitutive expression of the NT gene is regulated by a complex interplay between the proximal CRE/AP-1-like element and a region that binds orphan hormone receptor NR2F2 [573]. The C terminus of NR2F2 strongly represses, and the N-terminal domain antagonizes, the transcription of the NT gene [574]. Furthermore, various cis-regulatory motifs in the proximal 120 bp of the 5′-flanking sequence are required for the constitutive expression of the NT gene [573,575] (Figure 12a).

#### 4.11.2. Metabolism and Signaling

NT is derived from precursor protein preproneurotensin following excision by prohormone convertases [572]. NT-NTR1 interaction induces intracellular signaling through PLC and the inositol phosphate signaling pathways, as well as through the MAPK pathway inhibition of Akt activity [577].

#### 4.11.3. Immunomodulation and Inflammation

Studies have shown that NT can modulate pain transmission both as a facilitator and an inhibitor [578,579]. Furthermore, NT can also modulate the immune response by stimulating cytokine synthesis and immune cells chemotaxis. NT can exert its anti-inflammatory effects by suppressing the release of pro-inflammatory cytokines in macrophages, as well as the downregulation of pro-inflammatory signaling pathways NF-κB and JNK in dendritic cells [580]. In addition, NT inhibits the expression of the cytokines IL-6, TNF-α, IL-10, and VEGF and upregulates the ERK pathway [581]. 

#### 4.11.4. Role of NT in the Cornea

NT receptors localize both in the cornea and the trigeminal ganglia [582], suggesting that NT and its analogues may be involved in antinociceptive functions in the cornea. Furthermore, the administration of NT analogues induces analgesic effects with minimal effects on corneal epithelial cell function at therapeutic doses [582].

## 5. Neuropeptides as Therapeutic Targets/Drugs for Corneal Diseases

As discussed above, neuropeptides in the cornea play an important role in immunomodulation and inflammation, and their levels are altered in ocular injuries/diseases (Table 3). The targeting of these neuropeptides and their receptors has been shown to have therapeutic benefits in corneal diseases. 

### 5.1. Corneal Wound Healing

SP is the most well-studied neuropeptide in corneal wound healing as it enhances epithelial cell migration, adhesion, and the phosphorylation of cytoskeletal proteins [583]. Several clinical trials have been carried out using topical SP that show promising results, in that treatment with SP promoted re-epithelialization and wound healing in persistent corneal epithelial defects [584], spontaneous chronic corneal epithelial erosion [585], and neurotrophic keratopathies [586,587]. The topical application of SP has been reported to promote diabetic corneal epithelial wound healing by improving mitochondrial function and ROS scavenging capacity via SP/NK-1R signaling [287]. However, in a rabbit model, eyedrops containing both SP and IGF-1 need to be used to affect the promotion of corneal epithelial wound healing [583,588,589]. SP/IGF-1 application also improves the epithelial barrier function in animal models of neurotrophic keratopathy where SP is deficient [48,588,590,591,592]. In order to prevent the miosis induced by full-length SP, a short FGLM-amide sequence derived from the C-terminal of SP has been used along with IGF to stimulate corneal epithelial cell migration and enhance wound healing [593,594].

### 5.2. Dry Eye Disease (DED)

DED is a multifactorial disorder of the ocular surface characterized by chronic inflammatory features. The pathogenesis and progression of DED also involves an enhanced SP release from sensory terminals, which promotes pathological corneal lymphangiogenesis, Treg dysfunction, the maturation and activation of antigen-presenting cells, and induces the Th17 phenotype in the ocular surface [595]. Various studies in animal models of DED have shown that the administration of NK1R antagonist can suppress these mechanisms and reduce the severity of DED. In a desiccating stress-induced mouse model of DED, the blockade of SP/NK1R signaling with spantide I significantly reduces corneal neovascularization [596]. In another mouse model of DED using a controlled environment chamber, the topical application of NK1R antagonists CP-99,994 and L-733,060 reduced the clinical signs of DED by suppressing MHCII expression via antigen-presenting cells and by reducing Th17 cell activity [597]. In a similar model, NK1R antagonist L733,060 also inhibited pathological corneal lymphangiogenesis by suppressing VEGF-C, VEGF-D, and VEGF receptor-3 in the cornea [294]. Furthermore, treatment with spantide I effectively restores Treg function and suppresses pathogenic Th17 response [598,599].

Neuropeptides such as CGRP and NPY are reduced in the tears of DED patients and are associated with the severity of the disease [339]. Given that the loss of the PACAP gene in mice causes dry eye-like symptoms, such as corneal keratinization and tear reduction, PACAP eyedrops can stimulate tear secretion by increasing the water permeability of lacrimal acinar cells through aquaporin 5 (AQP5) [190]. 

The application of α-melanocyte-stimulating hormone (α-MSH) twice a day to the ocular surface of a scopolamine-induced, aqueous-deficient dry eye model in rats improves tear secretion, tear film stability, and corneal integrity; restores the number and size of conjunctival goblet cells; and corrects overexpression of proinflammatory factors such as TNF-α, IL-1β, and IFN-γ [522].

### 5.3. Infectious Keratitis

Herpes simplex virus type 1 (HSV-1) keratitis is associated with higher levels of SP in the corneal stroma, and the subconjunctival administration of spantide I significantly reduces IL-6 and CCL3 proteins and the influx of neutrophils and CD4 T cells, leading to reduced corneal opacity and angiogenesis [295].

SP is also associated with the increased severity of Pseudomonas aeruginosa keratitis [231,600], and the systemic administration of SP antagonists can control the inflammation, possibly through the early apoptosis of immune cells and the downregulation of TNF-α, IL-1β, IL-18, and MIP-2 [231,388,600,601].

The application of exogenous VIP also promotes healing in experimental Pseudomonas aeruginosa keratitis by regulating pro- and anti-inflammatory cytokines, growth factors, and Toll-like receptors [389].

### 5.4. Corneal Neovascularization

SP production increases after injuries to the corneal epithelium from alkali burns and in suture-induced corneal neovascularization animal models [285]. The administration of NK1R antagonists (Lanepitant and Befetupitant) can reduce corneal SP levels and leukocyte infiltration, leading to reduced corneal neovascularization [284,285]. A recent work suggests that the NK1R antagonist Fosaprepitant can inhibit pain transmission by decreasing SP release in the tear fluid and in the TG [276]. Furthermore, SP also mobilizes CD29^+^ stromal cells from the bone marrow to the injured tissue to accelerate wound healing in an alkali-burn model of mouse and rabbit eyes [602]. 

### 5.5. Corneal Transplantation

Significant amounts of SP impair Treg functions necessary for an allograft’s survival, and SP antagonists can restore corneal immune privilege [603]. Studies have shown that corneal allograft survival can be improved via the local application of the neuropeptides VIP [604] and α-MSH [519]. It has been shown that VIP effectively maintains endothelial cell integrity post-transplantation [604], and α-MSH can decrease inflammatory cell influx into the graft site, suppressing the delayed-type hypersensitivity response in hosts [519] using a mouse model of allogeneic corneal transplantation.

## 6. Conclusions

The neuropeptide modulation of immune cells is quite complex, and at times, studies have found pro- and anti-inflammatory properties for the same neuropeptide. While in some cases these differing effects may be due to differences in the expression of neuropeptide receptor isoforms and/or cell-type specific differences in downstream effectors, a further nuance to consider is that of the microenvironment. Likely, the ultimate effects of neuropeptide signaling are influenced by the presence of additional neuropeptides, cytokines, and the rest of the microenvironment. However, despite these gaps in knowledge, which warrant further study, there remains great promise for the potential to harness this signaling in the development of new therapies. Thus, the therapeutic effects and treatment outcomes of neuropeptides warrant the translation of the animal studies into clinical trials for the development of novel and effective interventions for ocular surface injuries and diseases.

## Figures and Tables

**Table 1:** Neuropeptide structures and their receptors.

Neuropeptides	Sequence	Receptors and Relative Affinity	References
Substance P	RPKPQQFFGLM	NK1R-F (Full) > NK1R-T (Truncated) >> NK2R, NK3R	[71,72,73,74,75,76,77]
CGRP	ACDTATCVTHRLALLSRSGG-VVKNNFVPTNVGSKAF	CLR/RAMP1 >> CLR/RAMP2 ≈ CLR/RAMP3	[78,79,80,81,82,83]
Adrenomedullin	YRQSMNNFQGLRSFGCRFGTCTVQKLAHQIYGFTDKDKDNVAPRSKISPQGY	CLR/RAMP2 ≈ CLR/RAMP3 >> CLR/RAMP1	[84,85,86]
VIP	HSDAVFTDNYTRLRKQMAVKKYLNSILN	VPAC1R > VPAC2R >> PAC1R	[87,88,89,90,91,92,93,94,95]
PACAP	HSDGIFTDSYSRYRKQMAVKKYLAAVLGKRYKQRVKNK	PAC1R >> VPAC1R ≈ VPAC2R	[87,96,97,98,99,100,101,102,103]
NPY	YPSKPDNPGEDAPAEDMARYYSALRHYINLITRQRY	Y1 ≈ Y2 ≈ Y5 >> Y4	[104,105,106,107,108,109,110,111,112,113,114]
SST	SANSNPAMAPRERKAGCKNFFWKTFTSC	SST2 ≈ SST3 ≈ SST5 > SST1 ≈ SST4	[115,116,117,118,119]
α-MSH	SYSMEHFRWGKPV	MC1R ≈ MC3R > MC4R > MC5R	[120,121,122,123,124,125,126]
Galanin	GWTLNSAGYLLGPHAVGNHRSFSDKNGLTS	GAL1R ≈ GAL2R > GAL3R	[127,128,129,130,131,132,133,134,135,136,137,138]
Opioid Growth Factor (OGF)/Met-enkephalin	YGGFM	μ >> OGFR > δ >> κ	[139,140,141,142,143,144]
Neurotensin	QLYENKPRRPYIL	NTS1R ≈ NTS2R	[145,146,147,148,149,150,151,152,153]

**Table 2:** Expression of neuropeptides and their receptors on the ocular surface.

Neuropeptides (Tissue or Fluid)	Receptors (Tissue or Fluid)	References
SP (nerve fibers in corneal epithelium and stroma, normal tears)	NK1R (native and cultured corneal epithelial cells, mast cells, T cells, monocytes, conventional dendritic cells, and Langerhans cells)	[10,154,169,170,171,172,173,174,175,176,177]
CGRP (nerve fibers in corneal epithelium and stroma, normal tears)	CLR/RAMP1 (corneal and limbal epithelial cells, T cells, innate lymphoid cells, macrophages, conventional dendritic cells)	[158,159,169,178,179,180,181,182,183,184]
Adrenomedullin (corneal nerves)	CLR/RAMP2, CLR/RAMP3 (Corneal epithelium, stroma, and endothelium; lymphatic and vascular endothelium; T cells, dendritic cells)	[185,186,187,188,189]
VIP (corneal nerves in anterior stroma)	VPAC1-R, VPAC2-R (lacrimal glands—basal side of acinar cells and ducts, T cells, monocytes)	[158,180,190,191]
PACAP (corneal nerves, tears, lacrimal gland nerves, and acinar cells)	PAC1-R, VPAC1-R, VPAC2-R (lacrimal glands—basal side of acinar cells and ducts, T cells, monocytes)	[87,190,191,192,193,194,195,196]
NPY (corneal nerves in anterior stroma)	Y1, Y2, Y4, Y5, and y6 receptors (T cells, monocytes, mast cells)	[158,161,162,163,164,165,166,197,198,199,200]
SST (lacrimal gland, corneal nerves)	SST1R-SST5R (meibomian gland, T cells, B cells, monocytes)	[158,201,202,203]
α-MSH (cornea)	MC1R-MC5R (corneal endothelial cells, acinar cells in lacrimal glands, T cells, B cells, NK cells, monocytes, granulocytes)	[69,204,205,206,207]
Galanin (corneal and conjunctival sensory nerves)	GalR1, GalR2, and GalR3 (NK cells, neutrophils, macrophages)	[156,158,208,209,210,211,212]
Opioid Growth Factor (OGF)/Met-Enkephalin (Corneal nerves, corneal epithelium)	OGFr (corneal epithelial cells)	[158,205]
Neurotensin (corneal nerves)	Neurotensin receptor (cultured human corneal keratocytes)	[165,167,168]

**Table 3:** Summary of neuropeptide functions.

Neuropeptide	Functions	References
Substance P	Pro-inflammatory. Promotes macrophage and neutrophil phagocytosis, increases pro-inflammatory cytokine secretion, activates mast cells and NK cells, and enhances T cell proliferation. Promotes tear secretion and anti-apoptotic functions on corneal epithelial cells. May maintain stemness of limbal stem cells and promotes corneal wound healing. Promotes corneal angiogenesis and lymphangiogenesis, as well as leukocyte recruitment to the cornea during inflammation. Also has a chief role in pain.	[68,179,195,227,258,259,260,261,262,263,264,265,266,267,268,269,270,271,272,273,274,275,276,277,278,279,280,281,282,283,284,285,286,287,288,289,290,291,292,293,294,295,296,297,298]
CGRP	Causes vasodilation and is pro-inflammatory. Enhances the pro-inflammatory activity of lymphocytes, cDCs, and macrophages. Promotes corneal wound healing through effects on corneal epithelial cells. Its levels correlate with lacrimal gland function. Also has a role in pain.	[322,323,324,325,326,327,328,329,330,331,332,334,335,336,337,338,339]
Adrenomedullin	Promotes the proliferation of CD34+ progenitor cells and hematopoietic stem cells. Elevated levels in models of corneal inflammation. Knockdown diminishes corneal angiogenesis.	[187,341,344,360,361,362,363]
VIP	Pro- and anti-inflammatory effects that may be context- or receptor-dependent. Primes the oxidative burst response in neutrophils, and causes histamine release in mast cells. Inhibits production of inflammatory cytokines and increases IL-10 production. Enhances corneal wound healing and corneal allograft survival. Promotes corneal nerve regeneration by regulating neurotrophic factors. Promotes survival of corneal endothelial cells.	[67,364,376,377,378,379,380,381,382,383,384,385,386,387,388,389,390,391,392,393,394,395,396]
PACAP	Pro- and anti-inflammatory effects mediated in a context-dependent manner. Inhibits secretion of pro-inflammatory cytokines from macrophages. Involved in T cell maturation and can skew towards a Th2 phenotype. Regulates tear secretion and may have utility as a treatment for dry eye disease. Enhances corneal nerve regeneration and sensitivity and accelerates corneal wound healing.	[70,190,429,430,431,432,433,434,435,436,437,438]
NPY	Pro- and anti-inflammatory effects. Increases chemotaxis in various immune cells. Inhibits the maturation of cDCs and proliferation of T cells. Promotes pro-inflammatory cytokine release from macrophages. Enhances corneal angiogenesis through the Y2 receptor.	[462,463,464,465,466,467,468,469,470,471,472,473,474,475]
SST	Pro- and anti-inflammatory effects. Correlates with activation state of immune cells. Regulates lymphocyte migration and macrophage/monocyte phagocytosis. Demonstrated to have antiangiogenic properties, including inhibiting corneal neovascularization.	[201,488,489,490,491,492,493,494,495,496,497,498,499]
α-MSH	Anti-inflammatory effects with widespread suppression of inflammation. Inhibits pro-inflammatory cytokine production and immune cell chemotaxis. Promotes the induction of regulatory T cells. Improves survival of corneal allografts and enhances survival of corneal endothelial cells. Increases tear secretion and goblet cell function in dry eye disease.	[69,121,124,502,507,508,509,510,511,512,513,519,520,521,522,523]
Galanin	Modulates neutrophil and NK cell functions. Present in the tear film, although its precise role in healthy and diseased corneas remains unclear. Also involved in pain signaling.	[210,212,524,537,538,539,540,541,542,543,544,545,546,547]
OGF/ Met-Enkephalin	Immunomodulatory effects on many immune cells, such as inhibiting regulatory T cells, enhancing NK cell activity, and increasing phagocytosis. Effects may be dependent on the presence of a potent immune stimulus. Suppresses corneal wound healing.	[549,555,561,562,563,564,565,566,567,568,569]
Neurotensin	Pro- and anti-inflammatory effects. Enhances chemotaxis and may stimulate or inhibit cytokine synthesis. Involved in pain signaling and has analgesic effects on the cornea.	[578,579,580,581,582]

## Data Availability

Not applicable.
